# Neuromuscular strength, not sagittal-plane joint or modeled muscle–tendon kinematics, is associated with 50-m sprint performance in youth male sprinters

**DOI:** 10.3389/fphys.2026.1858508

**Published:** 2026-07-22

**Authors:** Han Zhou, Jicheng Yang, Zhiyuan Lin, Chumeng Zhou, Wei Bao Liang, Ruixuan Zhang, Dajun Xuan, Wei Liang, Wenbai Huang

**Affiliations:** 1 School of Physical Education, Jinan University, Guangzhou, China; 2 Guangdong Provincial Key Laboratory of Speed Capability Research, Su Bingtian Center for Speed Research and Training, Jinan University, Guangzhou, China; 3 Guangdong Ersha Sports Training Center, Guangzhou, China; 4 Department of Physical Education, Sun Yat-sen University, Guangzhou, China

**Keywords:** hamstring eccentric strength, hip flexor strength, markerless motion capture, musculoskeletal modeling, neuromuscular function, Pose2Sim, sprint performance, youth athletes

## Abstract

**Introduction:**

Maximum-velocity sprinting imposes extreme mechanical demands on the hamstring muscle–tendon unit, yet how neuromuscular function, sprint kinematics, and modeled muscle–tendon behavior jointly differentiate youth sprinters of varying performance levels remains poorly characterized. We investigated the associations among these factors and 50-m sprint performance in youth male sprinters.

**Methods:**

Fifty-six athletes (14–20 years) were recruited; 46 with valid 50-m times weretertile-split into faster (n = 16, 5.97 ± 0.09 s), intermediate (n = 15, 6.30 ± 0.09 s), and slower (n = 15, 6.77 ± 0.27 s) groups. NordBord hamstring eccentric strength, ForceFrame isometric strength, isometric mid-thigh pull, standing long jump, and nine flexibility measures were assessed. In 43 athletes, two-dimensional high-speed videography with deep-learning pose estimation yielded 129 kinematic variables, and OpenSim/Pose2Sim modeling quantified 2,480 muscle–tendon kinematic parameters across 20 lower-limb muscle groups. Between-group comparisons used ANOVA or Kruskal–Wallis tests with Benjamini–Hochberg false discovery rate (FDR) correction; cross-validated penalized regression (LASSO/ridge) identified independent predictors, with age as a covariate in sensitivity analyses.

**Results:**

All four 50-m segments showed complete three-group separation (all p < 0.001, η2 = 0.70–0.84). Hamstring bilateral peak eccentric force (η2 = 0.369), hip flexor isometric peak force (η2 = 0.316), and standing long jump (η2 = 0.417) showed large between-group differences. Because the faster group was significantly older, these associations were re-examined with chronological age (and an estimated maturity offset) as covariates and remained robust. In exploratory analysis, a larger popliteal angle (poorer hamstring extensibility) was associated with slower times but did not survive age adjustment. Penalized regression retained standing long jump, hip flexor isometric force, and hamstring eccentric force among the leading predictors of 50-m time (LASSO leave-one-out R2 = 0.44). Of 129 kinematic variables, only 10 (all spatiotemporal) reached FDR significance; none of the 44 joint angles, 16 angular velocities, 53 inter-segmental coordination measures, or 2,480 muscle–tendon parameters survived correction.

**Discussion:**

These findings indicate that hip flexor and hamstring eccentric strength, rather than joint or muscle–tendon kinematics, are the key neuromuscular discriminators of youth sprint performance, with differences at maximum velocity expressed through temporal force application during ground contact rather than joint- or muscle-level motion.

## 1 Introduction

Maximum-velocity sprinting represents one of the most demanding mechanical tasks performed by the human musculoskeletal system. During the late swing phase of each sprint stride, the biarticular hamstring muscles undergo rapid active lengthening while generating large forces to decelerate the swinging shank, before transitioning to a concentric or quasi-isometric role to extend the hip during ground contact. Musculoskeletal-model-based simulations have estimated peak musculotendon strains in the long head of biceps femoris (BFlh) of approximately 12% and peak forces in semimembranosus exceeding nine times body weight at top sprinting speed (Schache et al., 2012; Chumanov et al., 2011). These extreme demands position the hamstring complex as both a critical determinant of sprint performance and the most frequently injured muscle group in track athletes, with lifetime injury prevalence reaching 95.6% in elite sprinters (Edouard et al., 2023).

The functional capacity of the hamstrings is most commonly assessed through maximal eccentric strength during the Nordic hamstring exercise, quantified using instrumented devices such as the VALD NordBord. A growing body of evidence links Nordic hamstring eccentric force to short-distance sprint performance: Markovic et al. (2020) reported a correlation of r = −0.52 between body-mass-normalized Nordic strength and 20-m sprint time in youth soccer players, and Ishøi et al. (2019) demonstrated that early-phase rate of force development (100 ms) was significantly associated with 0–30 m acceleration. Complementary measures of lower-limb neuromuscular function, including isometric mid-thigh pull (IMTP), standing long jump, and isometric hip and knee strength assessed via instrumented frames, have likewise been linked to acceleration and maximum-velocity capacities (Suchomel et al., 2016; Loturco et al., 2018). However, the overwhelming majority of this literature has been generated in adult elite or sub-elite cohorts, and comparatively little is known about how these neuromuscular characteristics differentiate youth sprinters of varying performance levels, particularly during the developmental window in which both anthropometric and neuromuscular maturation are still ongoing.

Beyond static functional assessment, advances in markerless motion capture and musculoskeletal modeling have made it increasingly feasible to quantify *in vivo* muscle–tendon behavior during high-speed sprinting in field settings. Deep-learning-based 2D pose estimation pipelines now permit accurate kinematic reconstruction without skin markers (Mathis et al., 2018; Pagnon, 2024), and OpenSim-based musculoskeletal modeling can in turn estimate musculotendon length and shortening velocity in individual lower-limb muscles across the gait cycle (Delp et al., 2007; Seth et al., 2018; Pagnon et al., 2021). Yet despite two decades of modeling research describing hamstring behavior during sprinting in adults, virtually no studies have applied these techniques to youth athletes, and it remains unclear whether differences in sprint performance among developing athletes are accompanied by detectable differences in joint kinematics or in modeled muscle–tendon kinematics, or whether such differences are confined to spatiotemporal gait variables.

A further unresolved question concerns the role of joint flexibility. The traditional view holds that greater hip and knee range of motion (ROM) facilitates sprinting through longer step length and more compliant force transmission. More recent biomechanical frameworks, however, suggest that the hamstring complex during sprinting operates through a combination of spring-driven (isometric), brake-driven (eccentric), and motor-driven (concentric) strategies, in which adequate musculotendon stiffness is essential for elastic energy storage and return (Kalkhoven et al., 2023). Under this view, excessive ROM accompanied by reduced stiffness could plausibly impair, rather than enhance, sprint performance. Empirical data testing this prediction in youth athletes are scarce.

Taken together, three gaps motivate the present study. First, no integrated dataset has simultaneously characterized hamstring eccentric strength, isometric joint strength, lower-limb power, and joint flexibility in youth male sprinters of clearly differentiated performance levels. Second, it is unknown whether between-level performance differences during the maximum-velocity phase manifest in joint kinematics, in modeled muscle–tendon kinematics, in spatiotemporal gait variables, or in some combination thereof. Third, the relationship between joint flexibility and sprint performance in this population has not been empirically tested against the stiffness-based framework. The present study therefore aimed to (i) compare hamstring eccentric strength, isometric strength, lower-limb power, and joint flexibility among youth male sprinters classified into faster, intermediate, and slower performance groups; (ii) identify independent predictors of 50-m sprint time using cross-validated penalized multiple regression; and (iii) characterize maximum-velocity sprint kinematics and OpenSim-derived lower-limb muscle–tendon kinematics across performance levels using markerless motion capture and musculoskeletal modeling. We hypothesized that faster sprinters would exhibit greater neuromuscular strength but that performance-level differences during maximum-velocity running would be more apparent in spatiotemporal than in joint-kinematic or muscle–tendon kinematic variables.

## 2 Materials and methods

### 2.1 Participants

Fifty-six male youth sprinters aged 14–20 years were recruited from regional sport schools and track-and-field training centers in Guangdong Province, China. Inclusion criteria were: (i) male sex; (ii) age 14–20 years; (iii) at least one year of formal sprint training; (iv) current participation in regular sprint training (≥3 sessions per week); and (v) freedom from lower-limb musculoskeletal injury in the preceding three months. Of the 56 participants assessed, 46 produced valid 50-m sprint times under the standardized testing protocol (see Section 2.3) and were included in the primary between-group analyses (of the 10 not retained, 6 did not complete the 50-m sprint test and 4 had incomplete data preventing inclusion in the merged dataset; this attrition was unavoidable given the demands of completing the full three-day testing protocol in an adolescent sample). A subsample of 43 athletes additionally completed the maximum-velocity high-speed videography session, and a complete-case subset of 42 athletes was available for the penalized regression analysis; complete-case n differed across models (42–46) because each model required a different set of predictors.

Tertile classification by 50-m time provides a balanced, interpretable contrast of performance levels of the kind commonly used in sprint and strength research; it is employed here as a descriptive framework for visualizing group differences, whereas the continuous correlation and cross-validated regression analyses serve as the primary inferential tests, since categorizing a continuous outcome reduces statistical power. Participants were classified into three performance groups based on tertile splits of their valid 50-m sprint times: a *faster* group (n = 16, 5.97 ± 0.09 s), an *intermediate* group (n = 15, 6.30 ± 0.09 s), and a *slower* group (n = 15, 6.77 ± 0.27 s).

This study was approved by the Medical Ethics Committee of Jinan University (approval number: JNUECKY-20260325-057) and conducted in accordance with the Declaration of Helsinki. Participants aged 18 years or older provided written informed consent for themselves; for participants under 18 years of age, a legal guardian provided written informed consent and the participant provided written assent prior to participation, after the testing procedures, potential risks, and right to withdraw had been fully explained.

### 2.2 Study design and overview

**Figure 1:**
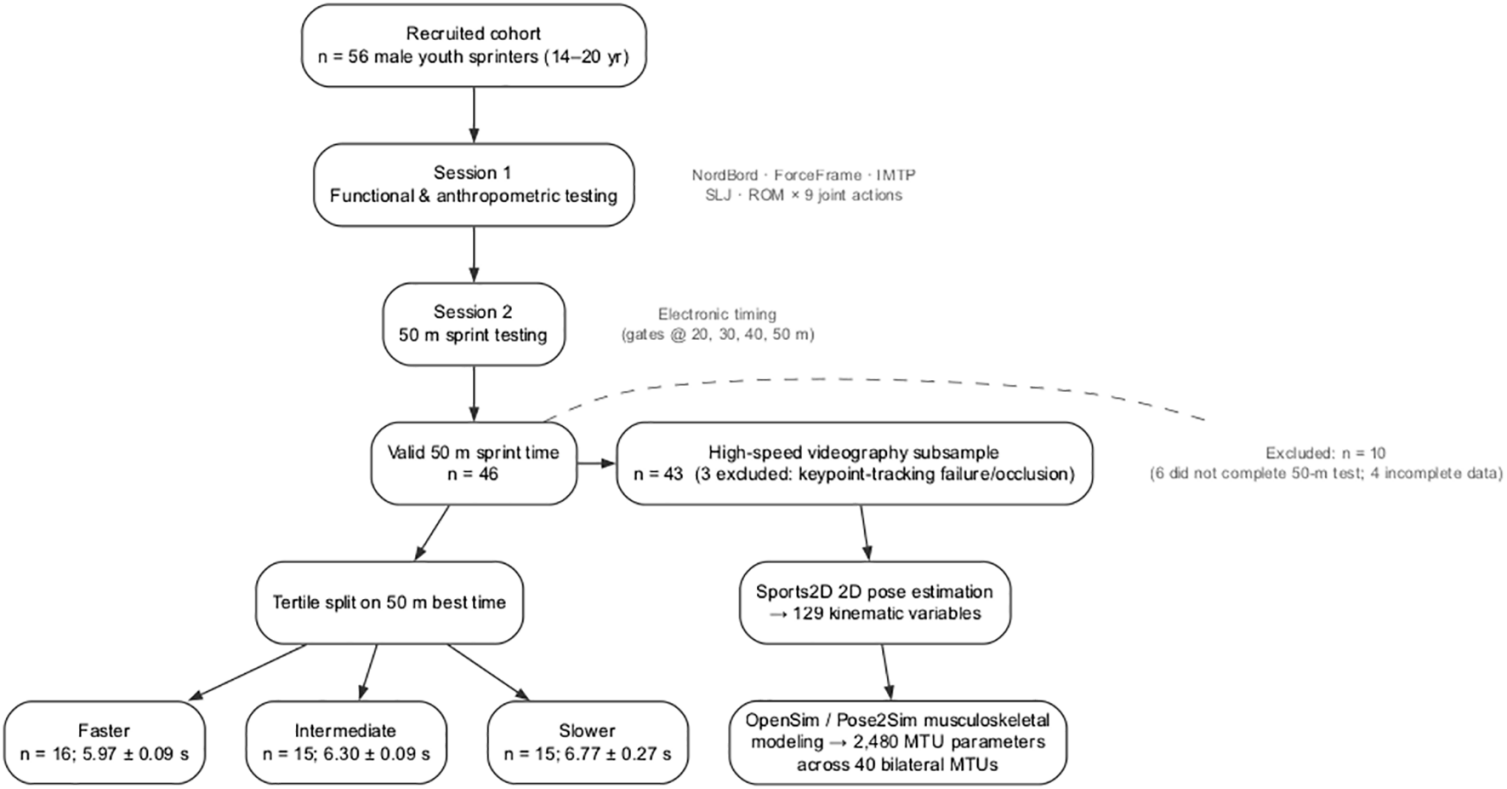
Study flowchart. STROBE-style flow diagram showing participant flow from recruitment through eligibility and classification into performance groups. Fifty-six male youth sprinters (14–20 years) were recruited from regional sport schools and training centers in Guangdong Province. Of these, 6 did not complete the 50-m sprint test and 4 had incomplete data that prevented inclusion in the merged analysis dataset, reflecting uncontrollable attrition in an adolescent field sample tested across three days, leaving 46 athletes with valid 50-m times, who were classified by tertile split into faster (n = 16), intermediate (n = 15), and slower (n = 15) groups. From these 46, two non-nested analysis branches followed: a subsample of 43 athletes with usable maximum-velocity video underwent markerless pose estimation and OpenSim musculoskeletal modeling, and a complete-case subset of 42 athletes entered the penalized regression analysis.

A cross-sectional comparative design was employed (**Figure 1**). All participants completed two testing sessions on separate days, with at least 48 hours between sessions and no strenuous training in the 24 hours preceding each session. Session 1 included anthropometric measurement, joint flexibility assessment, and neuromuscular strength testing (NordBord, ForceFrame, IMTP, standing long jump). Session 2 comprised the 50-m sprint test, with high-speed videography of the maximum-velocity phase performed within the same session for the kinematic subsample. All testing was conducted under controlled indoor conditions at a constant ambient temperature, with the exception of the 50-m sprint test, which was performed on an outdoor synthetic track under conditions of negligible wind (absolute wind speed verified to be < 2 m/s with an anemometer mounted on a tripod at the start line, oriented in the direction of sprinting, immediately before each run; continuous wind speed and direction were not recorded).

### 2.3 Anthropometric measurement and 50-m sprint test

Standing height was measured to the nearest 0.1 cm using a stadiometer, and body mass to the nearest 0.1 kg using a calibrated digital scale, with participants barefoot and wearing light athletic clothing. Age was recorded to the nearest 0.1 year from date of birth.

The 50-m sprint test was performed from a standing start on a standard outdoor synthetic track. Following a standardized warm-up consisting of 10 minutes of light jogging, dynamic stretching, and three submaximal acceleration runs of progressively increasing intensity, participants completed two maximal 50-m sprints separated by at least 6 minutes of passive recovery. Sprint times were recorded using a four-segment infrared photoelectric timing system (Smart Sports Equipment Factory, China) with timing gates positioned at 0, 20, 30, 40, and 50 m, yielding four split intervals (0–20, 20–30, 30–40, 40–50 m) in addition to total time. The faster of the two trials was retained for analysis. Trials in which participants showed obvious starting errors or visible deceleration before the finish line were repeated after additional rest.

### 2.4 Hamstring eccentric strength (NordBord)

Maximal eccentric hamstring strength was assessed using the VALD NordBord Hamstring Testing System (VALD Performance, Brisbane, Australia), following manufacturer guidelines. Participants knelt on the padded board with the ankles secured by individually instrumented ankle hooks containing uniaxial load cells. With hands crossed over the chest and trunk maintained in a neutral position, participants were instructed to lower themselves forward as slowly as possible by resisting the falling motion with their hamstrings, until they could no longer control the descent. Three maximal repetitions were performed after two submaximal familiarization trials, with 60 s of rest between repetitions. Recorded variables included bilateral peak force (N), bilateral mean force (N), body-mass-normalized peak force (N/kg), and left–right force asymmetry (%). The mean of the three valid repetitions was used for analysis.

### 2.5 Isometric strength (ForceFrame and IMTP)

Isometric hip and knee strength were assessed bilaterally using the VALD ForceFrame Strength Testing System (VALD Performance, Brisbane, Australia), following the manufacturer’s standardized test protocols (https://support.vald.com/hc/en-au/sections/4997742277401). Four positions were tested: hip flexion (standing; crossbar rotation 90°, flat paddle; one foot in front of the crossbar and the other behind with the dorsum of the foot resting on the pad; knees pointing forward), hip extension (standing; crossbar rotation −90°; bottom of the calf against the pad; leg held straight), knee flexion (standing; crossbar rotation 0°; knee flexed to 90° with the bottom of the calf resting on the pad), and knee extension (seated; crossbar rotation 0°; knee flexed to 90°). For each position, participants performed two 5-second maximal isometric contractions with 30 s of rest between trials, and the higher peak force (N) was retained for each side. The device records peak force for the left and right limbs separately; the bilateral value used for analysis was the mean of the left and right peak forces.

Isometric mid-thigh pull (IMTP) was performed on a fixed power rack with a non-elastic strap connecting the bar to the floor. Bar height was individually adjusted to a position corresponding to the midpoint of the thigh with the participant standing upright, hips and knees in approximately 145° and 125° of flexion respectively. Following a standardized warm-up, participants performed two maximal pulls of approximately 5 seconds, with 2 minutes of rest between trials. Pulling force was transmitted through a rigid steel chain connecting the barbell to an S-type strain-gauge load cell (model DYLY-103; 300-kg capacity; rated output 2.0 mV/V; combined nonlinearity, hysteresis, and repeatability ≤ 0.03% of full scale; Bengbu Dayang Sensing System Engineering Co., China) anchored to a custom steel frame fixed to the floor, and was digitized at 1,000 Hz using a bridge-input module (NI-9237) on a CompactDAQ chassis (cDAQ-9174; National Instruments, USA). The load cell was calibrated against known masses spanning 20–200 kg prior to data collection. Peak force was defined as the single maximum absolute value of the calibrated, channel-summed force–time signal after the automatically detected force onset, read from the unsmoothed signal (a 5-ms moving average was used only for onset/offset detection, not for the peak value); the higher of the two trials was retained and additionally expressed relative to body mass.

### 2.6 Standing long jump

Lower-limb explosive power was assessed via the standing long jump. From a standing position behind a marked line with feet shoulder-width apart, participants performed a maximal horizontal jump using a countermovement and bilateral arm swing, landing on both feet. Jump distance was measured from the take-off line to the rearmost contact point of the heels, to the nearest 1 cm. Three trials were performed with at least 1 minute of rest between trials, and the longest valid jump was retained.

### 2.7 Joint flexibility

Nine joint flexibility measures were assessed by a single trained examiner to minimize inter-rater variability: passive hip flexion (straight-leg raise), passive hip extension (modified Thomas test), passive hip abduction, passive hip internal rotation, passive hip external rotation, the popliteal angle (90–90 active knee extension test; recorded as the residual deficit from full knee extension with the hip held at 90°, where a larger angle denotes poorer hamstring extensibility; Brosseau et al., 2001), passive knee flexion, passive ankle dorsiflexion (knee extended), and passive ankle dorsiflexion (knee flexed). All measurements were performed bilaterally using an aluminum goniometer (Changlai, China) following established protocols, with the mean of left and right sides used for analysis. Each measurement was repeated twice and the mean recorded.

### 2.8 Maximum-velocity sprint kinematics

A subsample of 43 participants underwent two-dimensional high-speed videography during the maximum-velocity phase of the 50-m sprint. Two high-speed industrial cameras (MV-CS016-10UC, Hikvision, China; 1440 × 1080 pixels, 6-mm lens), each recording a single sagittal-plane view, were positioned perpendicular to the running lane at the 35-m and 45-m marks, capturing the 30–40 m and 40–50 m segments of the sprint, respectively. Each camera was placed at a horizontal distance of 13.9 m from the lane centre and a mounting height of 1 m, with its optical axis aligned with the participant’s centre of mass at the moment the runner crossed the corresponding camera plane. The aperture was set to approximately f/4 to balance exposure under the outdoor lighting, and focus was fixed on the calibration frame to ensure image sharpness. Although these cameras support acquisition rates of up to 240 fps, outdoor lighting required reduced frame rates to maintain adequate exposure; the achieved acquisition rate therefore varied across recordings (mean 124 fps, range 120–136 fps), and the kinematics of each trial were computed using that trial’s exact frame rate rather than being resampled to a common nominal value. Calibration was performed independently for each camera before testing session using a rigid L-shaped reference frame of known dimensions (1.45 m × 1.40 m) placed at the lane centre in the plane of motion, perpendicular to each camera’s optical axis, from which a dual-axis pixel-to-metre scale was derived. The independent diagonal of the frame (2.016 m) was reconstructed to within 5.0 mm (0.25%), the horizontal and vertical scales were closely matched (ratio = 1.0025), and the calibrated field of view was 11.05 × 8.26 m. Because joint angles are scale-invariant, this scaling affects only absolute distances and absolute muscle–tendon lengths. All sprints were performed within a single standard lane (1.22 m wide) whose centre coincided with the calibration plane, and athletes ran along the lane centreline, so out-of-plane displacement was limited to approximately half the lane width.

Two-dimensional pose estimation was performed using Sports2D (v0.8.26; Pagnon, 2024) with the RTMPose body_with_feet model (HALPE-26 keypoint family), which outputs 23 anatomical keypoints in both pixel and metric coordinates per frame. Spatiotemporal variables (step length, step frequency, ground contact time, flight time, contact-to-flight ratio, and center-of-mass horizontal velocity) were derived for three to four consecutive steps within the maximum-velocity zone. Joint angles (hip, knee, ankle), angular velocities, and inter-segmental coordination measures were computed across the gait cycle. In total, 129 kinematic variables were extracted per participant.

### 2.9 Musculoskeletal modeling and muscle–tendon kinematics

Lower-limb muscle–tendon kinematics during the maximum-velocity sprint phase were estimated using OpenSim (v4.5.2; Seth et al., 2018) within the Pose2Sim framework (v0.10.40; Pagnon et al., 2021). A full-body musculoskeletal model (Model_Pose2Sim_muscles_flex.osim, internal name *Pose2Sim_WithMusclesAndConstraints*) was used, adapted from the model of Beaucage-Gauvreau et al. (2019) with knee joint angle definitions from Rajagopal et al. (2016) (the generic full-body musculoskeletal model distributed with Pose2Sim; Pagnon et al., 2022, combining the lifting-model spine definition and the gait-model knee definition). The model comprised 30 body segments, 30 joints, 62 generalized coordinates, and 318 muscle–tendon units. Relative to the original model, the pelvis was modeled as a free six-degree-of-freedom segment, a ball joint was added at the head–neck, forearm pronation/supination was locked, and the upper limits of hip flexion (150°), adduction (−60°), rotation (−70°), and knee flexion (155°) were extended to accommodate the extreme joint excursions observed during sprinting.

Each model was individually scaled to the participant’s height, body mass, and segment lengths using the segment-specific scaling routine within Pose2Sim. Prior to inverse kinematics, the two-dimensional keypoint trajectories were gap-filled by linear interpolation (gaps ≤ 10 frames), de-spiked with a Hampel filter (7-frame window, 2 SD), and low-pass filtered with a zero-phase fourth-order Butterworth filter (6 Hz cutoff) applied to the keypoint coordinates and to the derived joint and segment angles; because the slow-motion factor was set to 1.0, the cutoff was not rescaled. The filtered metric trajectories were used for all subsequent inverse-kinematics and muscle–tendon computations, with no additional filtering applied downstream. Inverse kinematics was solved by Pose2Sim using OpenSim’s least-squares marker tracking, mapping the 2D Sports2D keypoints onto the model’s generalized coordinates and restricted to the sagittal plane. The model’s default coordinate ranges were extended to prevent the solver from clamping at the extreme joint angles reached during sprinting; visual inspection of the resulting kinematics confirmed that this did not produce non-physiological joint configurations or muscle-path artefacts. Muscle–tendon unit length was then computed via forward kinematics along the path points of each muscle as the cumulative path length, and muscle–tendon velocity was obtained as the central-difference time derivative of length. Because no ground reaction force or electromyographic data were collected, no inverse dynamics, static optimization, or muscle force estimation was performed; analyses were therefore restricted to muscle–tendon kinematics, defined here as length and length-derived velocity along each muscle’s path.

Forty bilateral muscle–tendon units (20 anatomical lower-limb muscle units sampled on both the left and right sides) were analyzed across seven muscle regions: hamstrings (biceps femoris long head, biceps femoris short head, semimembranosus, semitendinosus), quadriceps (rectus femoris, vastus intermedius, vastus lateralis, vastus medialis), gluteus maximus (superior, middle, and inferior compartments), gluteus medius (anterior, middle, and posterior compartments), hip flexors (psoas, iliacus), triceps surae (medial gastrocnemius, lateral gastrocnemius, soleus), and tibialis anterior. For each of these 40 muscle–tendon units we extracted six event-specific lengths (at touchdown and toe-off of three consecutive steps, TD1–TD3 and TO1–TO3) together with five phase-wise summaries (peak length, minimum length, length excursion, peak lengthening velocity, and peak shortening velocity) within each of five step-specific sub-phases (stance 1, swing 1, stance 2, swing 2, stance 3), giving 31 quantities per muscle–tendon unit. Because each step alternates the support and swing limb, these series were tabulated under two complementary labelling schemes: by anatomical side (left/right) and by functional role (support/swing), so that 40 × 31 × 2 = 2,480 muscle–tendon kinematic parameters were obtained per participant. These two labelling schemes are not duplicates: the support/swing series are assigned per participant according to the stance limb rather than being a relabelling of the left/right columns, so that, because the stance limb varies across participants, the anatomically referenced (left/right) and functionally referenced (support/swing) quantities are numerically distinct. All 2,480 quantities were therefore retained as distinct tests in the false-discovery-rate correction. The full musculoskeletal model contained 318 muscle–tendon units; the 40 bilateral lower-limb units listed above were those carried into this analysis.

### 2.10 Reliability

Joint flexibility was assessed by a single examiner; intra-rater reliability of goniometric range-of-motion measurement is good to excellent for the protocols used (ICC > 0.90; Brosseau et al., 2001), and the duplicate-measurement protocol further limited within-session error. The device-based strength measures have well-established reliability, with good relative reliability reported for both the NordBord (ICC 0.86–0.89) and the ForceFrame (ICC 0.78–0.85) in adolescent athletes of comparable age (Ferguson et al., 2024). For the markerless kinematics and modeled muscle–tendon lengths, within-session reliability was quantified directly in the present sample (43 participants, 170 trials) as single-measurement, absolute-agreement intraclass correlation coefficients [ICC(2,1)] computed across the multiple maximum-velocity trials per participant. Modeled hamstring muscle–tendon peak lengths (biceps femoris long head, semitendinosus, and semimembranosus) showed excellent reliability (ICC 0.95–0.97; coefficient of variation < 1%), and ground contact time was good (ICC 0.78). Spatiotemporal step variables were more variable when pooled across the two analysis windows, but window-internal reliability was substantially higher (e.g., 40–50 m step length ICC 0.76 and step frequency 0.76), indicating that the pooled values were deflated by genuine between-window differences in gait rather than by measurement error. Flight time (ICC ≈ 0.27) and the modeled muscle–tendon lengthening velocities (ICC 0.32–0.39), both of which depend on numerical differentiation, were the least reliable and are interpreted with corresponding caution.

### 2.11 Statistical analysis

All statistical analyses were performed in Python (v3.13.3) using the *pandas*, *SciPy*, and *statsmodels* libraries for descriptive statistics, normality testing, parametric and non-parametric group comparisons, correlation analyses, and multiple linear regression. Raw data were imported from Excel files using *openpyxl*, and figures were generated with *matplotlib* and *seaborn*. Normality of distributions was assessed using the Shapiro–Wilk test and visual inspection of Q–Q plots. Because the Shapiro–Wilk test has limited power to detect departures from normality at the per-group sample sizes used here (n ≈ 14–16), this routing was treated as a practical guide rather than a strict decision rule. Descriptive statistics are presented as mean ± standard deviation for normally distributed variables and as median otherwise.

Between-group comparisons across the three performance groups were conducted using one-way analysis of variance (ANOVA) for normally distributed variables and the Kruskal–Wallis test for non-normally distributed variables. Effect sizes were reported as η^2^ for ANOVA and ϵ^2^ for Kruskal–Wallis, with thresholds of 0.01, 0.06, and 0.14 for small, medium, and large effects, respectively. Because η^2^ (ANOVA) and ϵ^2^ (Kruskal–Wallis) are derived on different bases, they are not strictly on the same scale; effect-size magnitudes are therefore compared only approximately across the two test families. *Post-hoc* pairwise comparisons used Tukey’s HSD or Dunn’s test with Bonferroni adjustment, as appropriate. For the high-dimensional kinematic (n = 129) and muscle–tendon kinematic (n = 2,480) variable sets, the Benjamini–Hochberg procedure was applied to control the false discovery rate (FDR) at q < 0.05.

Bivariate associations between functional, kinematic, and muscle–tendon variables and 50-m sprint times were examined using Spearman’s rank correlation coefficient. To identify independent predictors of 50-m sprint performance, penalized multiple linear regression (LASSO and ridge, with the penalty parameter selected by cross-validation) was performed with 50-m total time as the dependent variable; candidate predictors comprised the neuromuscular and flexibility variables that showed significant bivariate correlation (p < 0.05) together with chronological age. Coefficients were estimated on standardized predictors in complete cases; model performance was evaluated by leave-one-out cross-validation (LOOCV) and repeated k-fold cross-validation, and coefficient stability was assessed by bootstrap resampling (1,000 iterations) with selection frequencies reported. Variance inflation factors (VIF) were computed to assess multicollinearity.

To account for the potential confounding effect of chronological age given the 14–20 year age range and the well-documented influence of biological maturation on neuromuscular performance, analysis of covariance (ANCOVA) was conducted on all variables that reached significance in the primary between-group analyses, with age as a covariate.

Statistical significance was set at α = 0.05 (two-tailed) for all tests, with FDR-corrected q < 0.05 used for the high-dimensional kinematic and muscle–tendon kinematic variable sets.

## 3 Results

### 3.1 Participant characteristics

Of the 56 male youth sprinters initially recruited, 46 produced valid 50-m sprint times and were retained for the primary between-group analyses; a subsample of 43 also completed the maximum-velocity videography session. Participant characteristics by performance group are presented in **Table 1**. The three groups did not differ significantly in standing height (176.69 ± 4.35, 176.47 ± 5.45, and 175.07 ± 5.34 cm; p = 0.50), body mass (66.56 ± 4.95, 66.13 ± 5.48, and 64.75 ± 9.93 kg; p = 0.77), arm span, shoulder width, pelvic width, or lower-limb length. Chronological age, however, differed significantly across groups (19.25 ± 0.86, 17.93 ± 1.16, and 17.47 ± 1.25 years; Kruskal–Wallis H = 17.51, p < 0.001, ϵ^2^ = 0.361), with the faster group being older than both the intermediate (p < 0.01) and slower (p < 0.001) groups. This age imbalance motivated the ANCOVA sensitivity analyses reported in Section 3.7.

**Table 1:** Participant characteristics by performance group.

Variable	Faster (n=16)	Intermediate (n=15)	Slower (n=15)	Test	p	Effect size	FDR p
Age (years)	19.25 ± 0.86	17.93 ± 1.16	17.47 ± 1.25	K–W	<0.001	ϵ^2^ = 0.361	0.001
Height (cm)	176.69 ± 4.35	176.47 ± 5.45	175.07 ± 5.34 ^a^	K–W	0.496	ϵ^2^ = −0.014	0.867
Body mass (kg)	66.56 ± 4.95	66.13 ± 5.48	64.75 ± 9.93 ^a^	ANOVA	0.767	η^2^ = 0.013	0.937
Arm span (cm)	177.85 ± 4.72	177.82 ± 6.11	175.37 ± 7.35	ANOVA	0.449	η^2^ = 0.037	0.867
Shoulder width (cm)	34.30 ± 1.58	34.05 ± 2.15	34.01 ± 2.30	ANOVA	0.912	η^2^ = 0.004	0.937
Pelvic width (cm)	23.96 ± 1.36	23.83 ± 1.62	24.87 ± 2.90	K–W	0.937	ϵ^2^ = −0.043	0.937
Lower-limb length (cm)	93.15 ± 3.09	94.35 ± 3.13	92.70 ± 4.75 ^a^	ANOVA	0.460	η^2^ = 0.035	0.867

Values are mean ± SD. K–W = Kruskal–Wallis test.

^a^One slower-group participant had missing data for height, body mass, and lower-limb length; n = 14 for these three variables.

### 3.2 50-m sprint segment times

**Figure 2:**
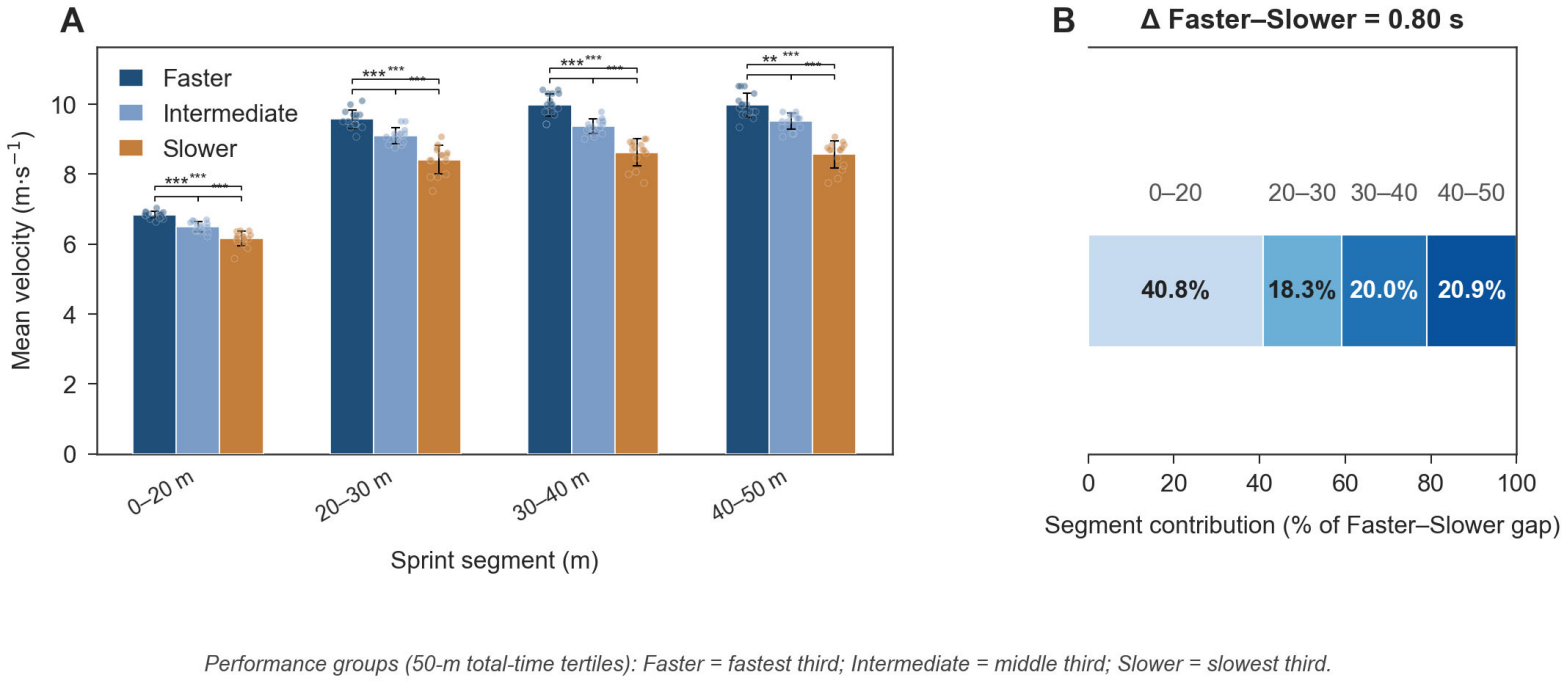
50-m sprint segment performance across performance groups. **(A)** Mean horizontal velocity within each 50-m segment (0–20, 20–30, 30–40, 40–50 m) for faster (n = 16), intermediate (n = 15), and slower (n = 15) groups, with error bars representing standard deviation. Asterisks indicate pairwise post-hoc significance (**p < 0.01; ***p < 0.001). **(B)** Relative contribution of each segment to the total between-group time difference. The 0–20 m acceleration phase accounted for the largest single share (40.8%), followed by the 40–50 m maintenance (20.9%), 30–40 m maximum-velocity (20.0%), and 20–30 m transition (18.3%) phases. All four segments showed complete three-group separation (all p < 0.001, η^2^/ϵ^2^ = 0.70–0.88).

50-m sprint segment times exhibited the most pronounced between-group separation observed in this study (**Table 2**; **Figure 2**). All four split intervals and the total time showed highly significant differences across the three groups (all p < 0.001), with effect sizes uniformly in the very large range (η^2^/ϵ^2^ = 0.70–0.88). Critically, *post-hoc* pairwise comparisons confirmed complete three-group separation across every segment: the faster, intermediate, and slower groups were each significantly different from one another in all four splits. This degree of three-way separation was not achieved by any other variable category in the study.

**Table 2:** 50-m sprint segment and total times by performance group (seconds).

Segment	Faster (n=16)	Intermediate (n=15)	Slower (n=15)	Test	p	Effect size	*Post-hoc* (G1/G2/G3)	FDR p
0–20 m (acceleration)	2.92 ± 0.04	3.08 ± 0.07	3.25 ± 0.12	K–W	<0.001	ϵ^2^ = 0.824	***/***/***	<0.001
20–30 m (transition)	1.04 ± 0.03	1.10 ± 0.03	1.19 ± 0.06	ANOVA	<0.001	η^2^ = 0.697	***/***/***	<0.001
30–40 m (max velocity)	1.00 ± 0.03	1.07 ± 0.02	1.16 ± 0.06	K–W	<0.001	ϵ^2^ = 0.837	***/***/***	<0.001
40–50 m (maintenance)	1.00 ± 0.03	1.05 ± 0.03	1.17 ± 0.06	K–W	<0.001	ϵ^2^ = 0.779	**/***/***	<0.001
0–50 m (total)	5.98 ± 0.09	6.30 ± 0.09	6.77 ± 0.27	K–W	<0.001	ϵ^2^ = 0.884	***/***/***	<0.001

Post-hoc pairwise comparisons: Faster vs Intermediate/Faster vs Slower/Intermediate vs Slower.

*p < 0.05, **p < 0.01, ***p < 0.001. K–W = Kruskal–Wallis test.

Since the performance groups were defined by total 50-m time, the following segment-level comparisons are reported descriptively rather than as independent inferential findings. Decomposing the between-group time difference by segment, the 0–20 m acceleration phase contributed the largest single share (40.8%), followed by the 40–50 m maintenance phase (20.9%), the 30–40 m maximum-velocity phase (20.0%), and the 20–30 m transition phase (18.3%). (Note: the sum of segment-level time differences slightly exceeds the difference in total times because the segment and total times were captured by independent photoelectric timing gates.) The acceleration phase thus accounted for approximately twice the share of any single later segment, indicating that performance-level differences are established earliest in the sprint and persist through the maximum-velocity and maintenance phases.

### 3.3 Neuromuscular strength and lower-limb power

**Figure 3:**
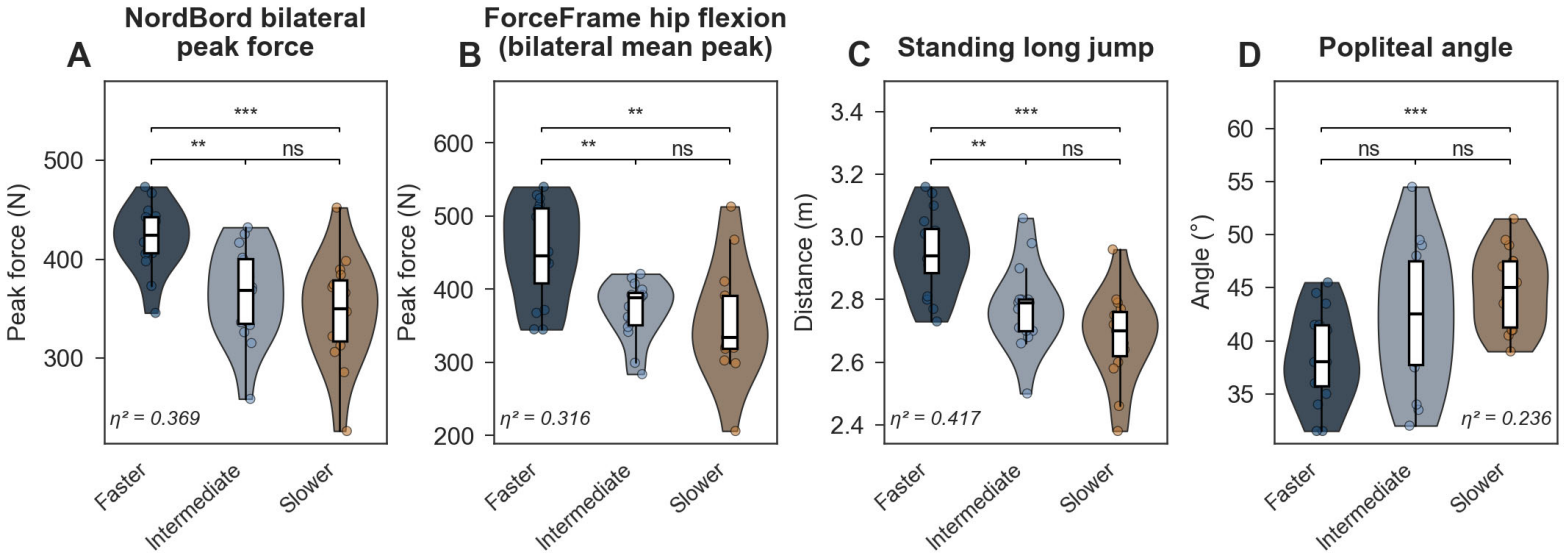
Neuromuscular strength and joint flexibility across performance groups. Violin plots with superimposed box plots and individual data points for the four variables with the largest between-group effect sizes in the neuromuscular and flexibility domains: **(A)** NordBord bilateral peak eccentric hamstring force (η^2^ = 0.369); **(B)** ForceFrame bilateral hip flexion peak isometric force (η^2^ = 0.316); **(C)** standing long jump distance (η^2^ = 0.417); **(D)** the popliteal angle (90–90 active knee extension test; η^2^ = 0.236). Because a larger angle denotes poorer hamstring extensibility, the slower group showed the largest popliteal angle and the faster group the smallest (greatest extensibility). All three strength variables retained significance after age-adjusted ANCOVA; the popliteal-angle effect did not (see **Table 5**) and is reported as exploratory.

Between-group comparisons of NordBord eccentric strength, IMTP, standing long jump, and ForceFrame isometric strength are summarized in **Table 3** and visualized in **Figure 3**.

**Table 3:** Neuromuscular strength, lower-limb power, and isometric strength by performance group.

Block	Variable	Faster (n=16)	Intermediate (n=15)	Slower (n ≤ 15)	Test	p	η^2^/ϵ^2^	FDR p
NordBord	Bilateral peak force (N)	410.09 ± 32.58	347.17 ± 46.05	334.90 ± 54.51	ANOVA	5.1 × 10^−5^	0.369	<0.001
	Bilateral mean force (N)	396.03 ± 32.40	328.19 ± 39.04	319.86 ± 57.05	ANOVA	1.8 × 10^−5^	0.398	<0.001
	Peak force/body mass (N/kg)	6.18 ± 0.49	5.26 ± 0.61	5.38 ± 0.87	ANOVA	6.6 × 10^−4^	0.295	0.005
	Force asymmetry (%)	5.52 ± 3.37	9.45 ± 8.74	7.31 ± 5.21	K–W	0.587	−0.022	0.640
	Time to peak force (s)	3.85 ± 1.83	3.31 ± 1.74	2.80 ± 1.48	ANOVA	0.235	0.065	0.352
Power	IMTP peak force (N)^b^	1564.62 ± 268.97	1322.56 ± 168.44	1319.45 ± 285.12	ANOVA	0.010	0.191	0.025
	Standing long jump (m)	2.95 ± 0.13	2.78 ± 0.13	2.68 ± 0.14	ANOVA	9 × 10^−6^	0.417	<0.001
ForceFrame^c^	Knee extension bilateral (N)	360.20 ± 65.17	274.94 ± 46.82	284.01 ± 59.99	ANOVA	2.8 × 10^−4^	0.329	0.007
	Knee flexion bilateral (N)	267.41 ± 59.20	205.92 ± 42.61	221.82 ± 50.77	ANOVA	0.005	0.221	0.023
	Hip extension bilateral (N)	797.55 ± 206.66	764.48 ± 157.56	789.21 ± 240.81	ANOVA	0.907	0.005	0.410
	Hip flexion bilateral (N)	435.27 ± 70.10	356.02 ± 37.21	344.37 ± 74.53	ANOVA	4.11 × 10^−4^	0.316	0.004
	Hip flexion/body mass (N/kg)	6.57 ± 1.14	5.42 ± 0.71	5.42 ± 1.07	ANOVA	0.002	0.253	0.007
	H:Q ratio	0.76 ± 0.16	0.75 ± 0.17	0.83 ± 0.22	ANOVA	0.445	0.039	0.494

Values are mean ± SD. Effect size: η^2^ for ANOVA, ϵ^2^ for K–W (Kruskal–Wallis).

^b^IMTP peak force (N), extracted from the force–time signal as described in the Methods; body-mass-normalized values are reported in the text.

^c^ForceFrame sample sizes vary (12–15 per group) because some participants did not complete all isometric positions.

NordBord rate-of-force-development impulses (50/100/150/200/250 ms) did not reach significance and are omitted from this table.

NordBord bilateral peak eccentric force differed significantly across groups (410.09 ± 32.58, 347.17 ± 46.05, 334.90 ± 54.51 N; ANOVA F = 12.55, p = 5.1 × 10^−5^, η^2^ = 0.369), as did bilateral mean force (396.03 ± 32.40, 328.19 ± 39.04, 319.86 ± 57.05 N; F = 14.21, p = 1.8 × 10^−5^, η^2^ = 0.398) and body-mass-normalized peak force (6.18 ± 0.49, 5.26 ± 0.61, 5.38 ± 0.87 N/kg; F = 8.77, p = 6.6 × 10^−4^, η^2^ = 0.295). All three measures fell into the large effect size range. By contrast, neither force asymmetry nor any of the rate-of-force-development impulse windows (50–250 ms) reached significance. *Post-hoc* comparisons consistently showed that the faster group differed from both the intermediate and slower groups, while the latter two did not differ from each other, a “faster-vs-the-rest” pattern that recurred throughout the strength variables.

Standing long jump distance showed the largest between-group effect of any neuromuscular variable (2.95 ± 0.13, 2.78 ± 0.13, 2.68 ± 0.14 m; F = 15.38, p = 9 × 10^−6^, η^2^ = 0.417). IMTP peak force was also significant (1564.62 ± 268.97, 1322.56 ± 168.44, 1319.45 ± 285.12 N; F = 5.08, p = 0.010, η^2^ = 0.191; body-mass-normalized 23.58 ± 4.03, 20.14 ± 3.10, 21.17 ± 4.01 N/kg, p = 0.040), again with the faster group separating from the other two.

ForceFrame isometric strength results paralleled the NordBord findings. Bilateral hip flexion peak force showed a large between-group effect (435.27 ± 70.10, 356.02 ± 37.21, 344.37 ± 74.53 N; F = 9.49, p = 4.11 × 10^−4^, η^2^ = 0.316), as did bilateral knee extension (360.20 ± 65.17, 274.94 ± 46.82, 284.01 ± 59.99 N; F = 10.05, p = 2.8 × 10^−4^, η^2^ = 0.329) and bilateral knee flexion (267.41 ± 59.20, 205.92 ± 42.61, 221.82 ± 50.77 N; F = 5.97, p = 0.005, η^2^ = 0.221). Bilateral hip extension peak force, in contrast, did not differ between groups (p = 0.91), nor did the hamstring-to-quadriceps (H:Q) ratio or hip flexor-to-extensor ratio.

### 3.4 Joint flexibility

Of the nine flexibility measures assessed, only the popliteal angle (90–90 active knee extension test) survived FDR correction (38.31 ± 4.31, 42.20 ± 6.62, 44.87 ± 3.77°; F = 6.65, p = 0.004, η^2^ = 0.236, FDR p = 0.027). The popliteal angle is the residual deficit from full knee extension with the hip flexed to 90°, a larger angle denotes poorer hamstring extensibility; the slower group therefore showed the largest popliteal angle (poorest extensibility) and the faster group the smallest (greatest extensibility). The association attenuated and lost significance after adjustment for chronological age (ANCOVA p = 0.11; Section 3.7) and is therefore reported as exploratory. Active knee-extension range of motion and the popliteal angle were obtained from separate tests and use opposite-direction scales: active knee-extension ROM is recorded as the achieved knee-extension angle (larger = greater extensibility), whereas the popliteal angle is the residual knee-flexion deficit in the 90/90 position (larger = poorer hamstring extensibility). The two are independent measures and should not be read as a single flexibility dimension. Active hip flexion range of motion (85.28 ± 11.21, 82.70 ± 9.05, 92.70 ± 7.75°; p = 0.017, η^2^ = 0.173) and active knee extension range of motion (159.22, 162.40, 166.00°; p = 0.023, ϵ^2^ = 0.129) followed monotonic patterns, although neither survived FDR correction in this section’s analysis. Hip extension, knee flexion, and ankle dorsiflexion range of motion did not differ between groups. Full results are provided in **Supplementary Table 1**.

### 3.5 Bivariate correlations and multiple regression

Spearman correlations between functional and kinematic variables and 50-m total time were computed for all 209 candidate variables; the complete ranked correlation table is provided in **Supplementary Table 2**. The strongest single correlate among the field-based functional measures was the standing long jump (ρ = −0.684, p < 0.001), followed closely by ForceFrame bilateral hip flexion peak force (ρ = −0.638), NordBord bilateral mean force (ρ = −0.637), and NordBord bilateral peak force (ρ = −0.610). IMTP peak force showed a weaker but significant association (ρ = −0.488). Among range-of-motion variables, the popliteal angle (ρ = +0.487) and active knee extension (ρ = +0.416) correlated positively with sprint time; for the popliteal angle, this indicates that poorer hamstring extensibility (a larger angle) accompanied slower performance. Segment-by-segment correlations (**Supplementary Table 3**) confirmed that these relationships were broadly consistent across all four 50-m intervals.

Because in-sample stepwise models substantially over-fit these data, residuals departed from normality (Shapiro–Wilk p = 0.005), four observations exerted high leverage (Cook’s distance), and the in-sample R^2^ greatly exceeded the cross-validated R^2^, independent predictors were instead identified using cross-validated penalized regression (**Table 4**; complete-case n = 42; 12 candidate predictors plus chronological age). The LASSO model (penalty α = 0.0252) achieved a leave-one-out cross-validated R^2^ of 0.446 (RMSE = 0.257 s), and ridge regression performed comparably (R^2^ = 0.436). Ranked by absolute standardized coefficient and bootstrap selection frequency, the leading predictors were standing long jump (β = −0.117; selected in 99% of bootstrap samples), ForceFrame bilateral hip flexion peak force (β = −0.081; 85%), NordBord bilateral peak force per body mass (β = −0.056; 74%), and active hip flexion range of motion (β = +0.071; 95%); the popliteal angle (90–90 active knee extension test) was retained with a small positive coefficient (β = +0.041; 78%). Chronological age was not retained by the LASSO (coefficient shrunk to zero; selection frequency 34%), indicating that the leading neuromuscular predictors were not merely proxies for age. A reduced field-based model (standing long jump, the popliteal angle, and NordBord bilateral mean force; n = 46) yielded a cross-validated R^2^ of approximately 0.398 (RMSE = 0.285 s) and is recommended for practical application. In-sample coefficients of determination (approximately 0.50–0.70) are reported in **Table 4** for reference only and should not be interpreted as the model’s expected predictive performance.

**Table 4:** Penalized (cross-validated) regression models predicting 50-m sprint total time.

Predictor	LASSO std. β	Ridge std. β	LASSO bootstrap 95% CI	Selection frequency	Retained by LASSO
Standing long jump	−0.117	−0.066	[−0.165, −0.028]	0.99	Yes
ForceFrame hip flexion (bilateral peak)	−0.081	−0.054	[−0.204, 0.000]	0.85	Yes
NordBord bilateral peak force/body mass	−0.056	−0.043	[−0.139, 0.000]	0.74	Yes
Active hip flexion ROM	+0.071	+0.048	[0.000, 0.129]	0.95	Yes
Popliteal angle (90–90 active knee extension)	+0.041	+0.040	[0.000, 0.110]	0.78	Yes
Passive knee extension ROM	+0.007	+0.027	[0.000, 0.069]	0.43	Yes
Active knee extension ROM	0	+0.030	[0.000, 0.084]	0.51	No
NordBord bilateral mean force	0	−0.019	[−0.111, 0.000]	0.29	No
NordBord bilateral peak force	0	−0.019	[−0.042, 0.000]	0.16	No
ForceFrame knee extension (peak)	0	−0.017	[−0.046, 0.000]	0.24	No
ForceFrame knee flexion (peak)	0	−0.003	[−0.049, 0.000]	0.30	No
IMTP peak force	0	+0.007	[−0.030, 0.133]	0.49	No
Age	0	−0.026	[−0.048, 0.000]	0.34	No

Dependent variable: 50-m sprint total time (s). Standardized coefficients from cross-validated penalized regression (complete-case n = 42; 12 candidate predictors plus chronological age). LASSO penalty α = 0.0252 (leave-one-out cross-validated R^2^ = 0.446, RMSE = 0.257 s); ridge α = 30.539 (R^2^ = 0.436). A reduced field-based model (standing long jump, the popliteal angle, and NordBord bilateral mean force; n = 46) yielded a cross-validated R^2^ of approximately 0.398 (RMSE = 0.285 s); in-sample R^2^ values (approximately 0.50–0.70) are over-optimistic and reported for reference only. Age was not retained by the LASSO. Selection frequency = proportion of 1,000 bootstrap resamples in which a predictor received a non-zero LASSO coefficient. Ordinary-least-squares diagnostics indicated over-fitting (Shapiro–Wilk p = 0.005; four high-leverage points).

Model A: R^2^ = 0.494, adjusted R^2^ = 0.469, F(2, 41) = 20.02, p < 0.001.

Model B: R^2^ = 0.496, adjusted R^2^ = 0.460, F(3, 42) = 13.78, p < 0.001. Model B was fitted as a field-deployable alternative excluding laboratory-grade ForceFrame variables.

### 3.6 Maximum-velocity sprint kinematics and muscle–tendon kinematics

**Figure 4:**
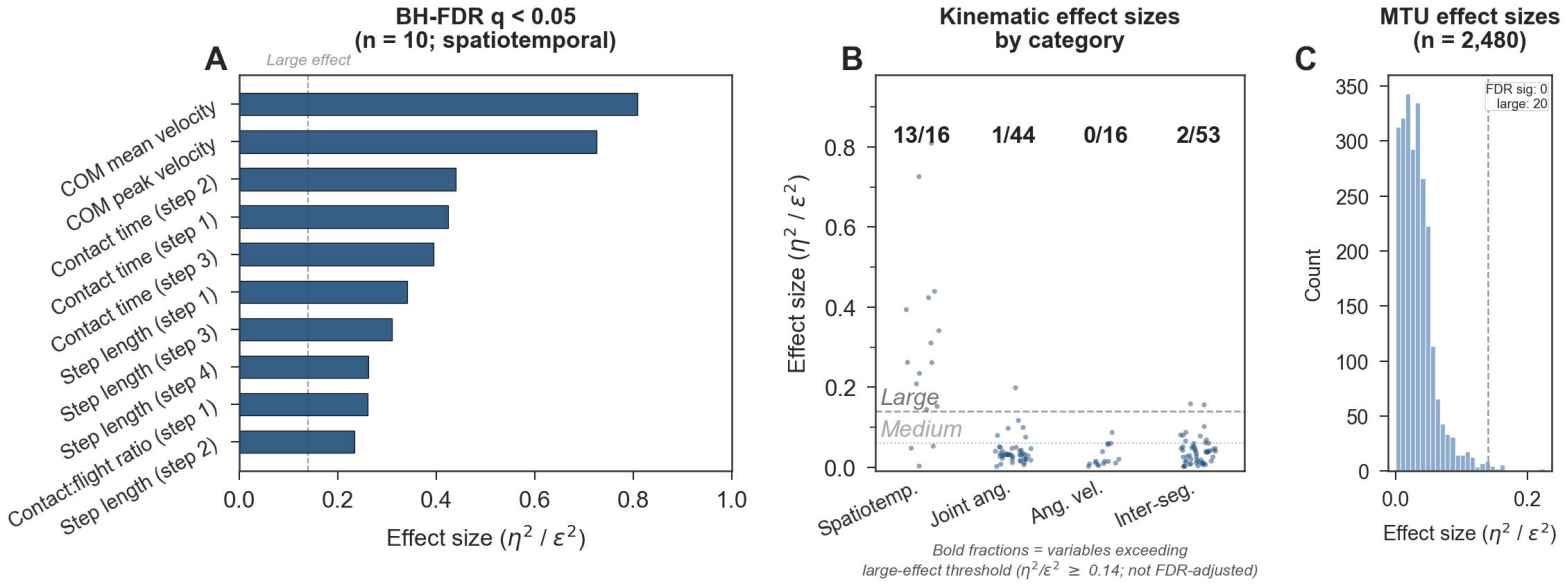
Maximum-velocity sprint kinematics and muscle–tendon kinematics. **(A)** Horizontal bar plot of the 10 kinematic variables that reached Benjamini–Hochberg FDR-corrected significance (q < 0.05). All 10 are spatiotemporal in nature: center-of-mass mean and peak velocity, ground contact time (three consecutive steps), step length (four consecutive steps), and contact-to-flight ratio (first step). The dashed vertical line indicates the threshold for a large effect (η^2^/ϵ^2^ = 0.14). **(B)** Effect-size strip plot across the four kinematic variable categories (spatiotemporal, joint angles, angular velocities, and inter-segmental coordination). Numbers above each column indicate the count of variables exceeding the large-effect threshold (η^2^/ϵ^2^ ≥ 0.14) out of the total tested: 13/16 for spatiotemporal, 1/44 for joint angles, 0/16 for angular velocities, 2/53 for inter-segmental coordination. Of these large-effect variables, only the 10 spatiotemporal variables survived Benjamini–Hochberg FDR correction (q < 0.05; Panel A); none of the joint-angle or inter-segmental variables with large raw effects survived correction. Horizontal reference lines mark the medium (0.06) and large (0.14) effect-size thresholds. **(C)** Distribution of effect sizes (η^2^/ϵ^2^) for all 2,480 OpenSim-derived muscle–tendon kinematic parameters; no parameter reached Benjamini–Hochberg FDR-corrected significance, and the maximum observed effect size was η^2^ ≈ 0.23. Only spatiotemporal variables discriminated the three performance groups; joint angles, angular velocities, inter-segmental coordination, and modeled lower-limb muscle–tendon kinematics were statistically indistinguishable across performance levels.

In the kinematic subsample (n = 43), 10 of the 129 extracted kinematic variables reached FDR-corrected significance and all 10 were spatiotemporal in nature (**Figure 4**). These comprised mean center-of-mass speed across the analyzed segment (10.55 ± 0.28, 9.98 ± 0.23, 8.99 ± 0.37 m/s; ϵ^2^ = 0.809), peak center-of-mass speed (10.93, 10.45, 9.46 m/s; ϵ^2^ = 0.726), ground contact times across three consecutive steps (η^2^/ϵ^2^ = 0.39–0.44, all faster-group < other groups), four consecutive step lengths (η^2^ = 0.23–0.34), and the contact-to-flight ratio of the first analyzed step (η^2^ = 0.261). None of the 44 joint angle variables (hip, knee, or ankle, at touchdown or toe-off), 16 angular velocity variables, or 53 inter-segmental coordination variables reached FDR significance. Full kinematic results are provided in **Supplementary Table 4**.

The musculoskeletal modeling pipeline yielded 2,480 muscle–tendon kinematic parameters across 20 lower-limb muscle groups (40 bilateral muscle–tendon units). After FDR correction, none of these 2,480 parameters reached significance (q ≥ 0.05 throughout). Even at uncorrected α = 0.05, only 31 raw p-values were < 0.05, corresponding to a raw hit rate of approximately 1.3%. Given the modest size of the kinematic sub-sample (n = 43), this null is interpreted as an absence of between-group differences detectable at this sample size rather than as positive evidence for equivalence of muscle–tendon kinematics across groups (see the minimum-detectable-effect and equivalence analysis, **Supplementary Table 6**). The breakdown by muscle group (**Supplementary Table 5**) showed that hamstrings (7/496 raw significant), quadriceps (9/496), gluteus maximus (4/372), gluteus medius (4/372), hip flexors (3/248), triceps surae (3/372), and tibialis anterior (1/124) all yielded null findings after correction. Similarly, all variable types, including peak shortening velocity, peak lengthening velocity, length range of motion, minimum length, support/swing ratios, and phase-specific length excursions, failed to discriminate the three performance groups.

### 3.7 Age-adjusted analyses

Because chronological age differed significantly across performance groups (Section 3.1), all variables that reached significance in the primary analyses were re-tested using ANCOVA with age as a covariate (**Table 5**). The neuromuscular results split into two patterns. NordBord bilateral peak force (adjusted p = 0.008), bilateral mean force (adjusted p = 0.002), body-mass-normalized peak force (adjusted p = 0.005), standing long jump (adjusted p = 0.002), ForceFrame bilateral hip flexion peak force (adjusted p = 0.021), ForceFrame bilateral knee flexion peak force (adjusted p = 0.024), ForceFrame bilateral knee extension peak force (adjusted p = 0.040), and active hip flexion range of motion (adjusted p = 0.019) all retained significance after age adjustment, indicating that the between-group differences in these variables were not driven by age alone. By contrast, IMTP peak force (adjusted p = 0.16), the popliteal angle (adjusted p = 0.11), and active knee extension range of motion (adjusted p = 0.88) lost significance after age adjustment, indicating that the between-group differences in these variables were largely attributable to the age imbalance. Hamstring and hip flexor strength thus emerged as the most robust neuromuscular discriminators of sprint performance, retained after adjustment for chronological age. The homogeneity-of-slopes assumption underlying these analyses of covariance was satisfied for all variables tested (all group × age interaction p > 0.12). Because chronological age is only a proxy for biological maturation, each athlete’s maturity offset (years from peak height velocity) was additionally estimated using the Mirwald equation (Mirwald et al., 2002), with sitting height derived as standing height minus measured lower-limb length; the faster group was significantly more mature than the slower group (4.2 versus 3.0 years past peak height velocity; Kruskal–Wallis p = 0.006). When this estimated maturity offset was substituted for chronological age as the covariate, all six core strength–performance associations remained significant, several were strengthened, indicating that the leading associations are not attributable to maturity differences alone.

**Table 5:** ANCOVA with chronological age as covariate: age sensitivity of the primary between-group findings.

Domain	Variable	F	p (age-adjusted)	Partial η^2^	p (unadjusted)	Status after age adjustment
Neuromuscular strength & power	NordBord bilateral peak force	5.39	0.008	0.204	<0.001	Retained
	NordBord bilateral mean force	7.31	0.002	0.258	<0.001	Retained
	NordBord peak force/body mass	6.14	0.005	0.230	<0.001	Retained
	IMTP peak force	1.91	0.160	0.083	0.010	Lost significance
	Standing long jump	7.19	0.002	0.255	<0.001	Retained
Joint flexibility	Hip flexion active ROM	4.36	0.019	0.172	0.017	Retained
	Knee extension active ROM	0.12	0.883	0.006	0.023	Lost significance
	Popliteal angle	2.36	0.107	0.101	0.003	Lost significance
ForceFrame isometric	Knee extension bilateral peak	3.50	0.040	0.149	<0.001	Retained
	Knee flexion bilateral peak	4.09	0.024	0.166	0.005	Retained
	Hip flexion bilateral peak	4.25	0.021	0.175	<0.001	Retained

Only variables that reached significance in the primary between-group analyses were re-tested.

“Retained” = age-adjusted p < 0.05; “Lost significance” = age-adjusted p ≥ 0.05.

## 4 Discussion

### 4.1 Hip flexor and hamstring strength as the primary neuromuscular discriminators

The principal finding of this study is that hip flexor isometric strength and hamstring eccentric strength are the most robust neuromuscular discriminators of 50-m sprint performance among youth male sprinters, and that these two strength qualities remain significant even after adjustment for chronological age. This positions hip flexor and hamstring strength not merely as correlates of sprint performance but as independent functional signatures of performance level in the developing athlete.

The hamstring eccentric strength findings that NordBord bilateral peak force ρ = −0.610, bilateral mean force ρ = −0.637 are consistent in magnitude and direction with previous reports in adult and youth male athletes. Markovic et al. (2020) reported r = −0.52 between body-mass-normalized Nordic hamstring strength and 20-m sprint time in youth soccer players, and Ishøi et al. (2019) found significant associations between early-phase rate of force development and 0–30 m acceleration in elite soccer players. Our results extend this evidence base in two important ways. First, by analyzing four separate 50-m segments, we show that the hamstring strength–performance relationship is preserved across the acceleration, transition, maximum-velocity, and maintenance phases, with correlation coefficients ranging from −0.43 to −0.64 (**Supplementary Table 3**). Second, the survival of NordBord measures through age-adjusted ANCOVA suggests that hamstring eccentric strength indexes a sprint-relevant neuromuscular capacity that is not simply a proxy for biological maturity.

The ForceFrame hip flexion finding is, to our knowledge, novel in this population. Hip flexion isometric peak force exhibited a large between-group effect (η^2^ = 0.316), survived FDR correction in the regression analysis, and emerged as one of the leading independent predictors of 50-m time in the penalized regression model (standardized β = −0.081; selected in 86% of bootstrap samples), second only to standing long jump. The biomechanical rationale is straightforward: during the swing phase of sprinting, rapid hip flexion repositions the recovering limb for the next ground contact, and Schache et al. (2012) showed that the iliopsoas and rectus femoris are among the most active hip flexors during this phase. Greater hip flexor strength likely enables both faster swing-leg recovery and a more aggressive forward repositioning of the foot at touchdown, mechanical features that align with the shorter ground contact times observed in the faster group (Section 3.6, Section 4.3).

### 4.2 The exploratory flexibility–performance relationship

A second principal finding and one that runs counter to widely held coaching assumptions concerns the exploratory flexibility associations. The variable that survived FDR correction was the popliteal angle (90–90 active knee extension test): the slower group showed the largest popliteal angle (poorest hamstring extensibility, 44.87° versus 38.31° in the faster group; η^2^ = 0.236), the angle correlated positively with 50-m time across segments (ρ = +0.42 to +0.52), and it was retained with a small positive coefficient in the penalized regression model. In other words, greater hamstring extensibility was associated with faster sprinting. Two cautions apply. First, the association lost significance after adjustment for chronological age (ANCOVA p = 0.11), so it may be confounded by the older, more trained profile of the faster group. Second, the sample is modest and the analysis exploratory. This flexibility association is therefore treated as hypothesis-generating only.

Notably, the direction of this exploratory association, greater hamstring extensibility in the faster group, runs counter to a simple tightness-is-beneficial reading of the stiffness-based framework of Kalkhoven et al. (2023), in which musculotendon stiffness aids elastic energy return. We therefore draw no training inference from it. Given the attenuation after age adjustment and the modest sample, the association is hypothesis-generating and would need to be tested directly, using validated extensibility and musculotendon-stiffness measures, before any training implication could be drawn.

It is essential to note that our cross-sectional design precludes any causal inference. Whether the slower athletes are slower because of their greater flexibility, or whether they happen to be more flexible because they have not yet undergone the training-induced stiffening typical of more advanced sprinters, cannot be resolved with these data. What can be said with confidence is that, in this cohort, greater flexibility was not protective of nor advantageous for sprint performance, a finding that should temper the prescription of aggressive flexibility training in youth sprint development.

### 4.3 Spatiotemporal, but not joint-kinematic or muscle–tendon kinematic, signatures of performance

The third major finding concerns the location and absence of performance-level differences during the maximum-velocity phase of sprinting. Of 129 kinematic variables extracted via deep-learning-based 2D pose estimation, only 10 reached FDR-corrected significance, and all 10 were spatiotemporal: center-of-mass velocity, ground contact time, step length, and the contact-to-flight ratio. None of the 44 joint angle variables, 16 angular velocity variables, or 53 inter-segmental coordination variables differentiated the three groups. Equally striking, none of the 2,480 OpenSim-derived muscle–tendon kinematic parameters across 20 lower-limb muscle groups survived FDR correction, with only 1.3% of raw p-values reaching nominal significance.

This pattern carries a clear message: at the speeds achieved by these youth sprinters, performance level differences are encoded in how briefly the foot contacts the ground and how far the body travels per stride, not in the angles, velocities, or modeled muscle lengths that the limb traces during that contact. Faster sprinters had ground contact times approximately 0.02 s shorter than slower sprinters across three consecutive steps (a 17–20% difference), and step lengths approximately 0.15–0.21 m longer (a 7–9% difference). These two parameters together capture the entire kinematic distinction between the groups; everything else including joint flexion angles, angular velocities, inter-segmental coordination, modeled muscle stretch and shortening velocity was statistically indistinguishable.

This finding aligns with the long-standing observation in adult sprint biomechanics that maximum-velocity sprinting differences are driven primarily by ground reaction force application during contact rather than by altered limb motion patterns (Weyand et al., 2000; Clark and Weyand, 2014). Faster runners produce greater mass-specific vertical force in less time, not by moving their limbs through different trajectories, but by applying force more rapidly during identical mechanical patterns. Our result is fully consistent with this “force-application-during-contact” model.

The OpenSim null finding warrants cautious interpretation. To be explicit about the scope of inference, we measured two-dimensional sagittal-plane joint kinematics and model-derived muscle–tendon lengths and velocities; we did not measure muscle forces, activations, tendon forces, joint moments, or three-dimensional and frontal- or transverse-plane mechanics; we can therefore conclude only that no between-group differences were detectable in the measured variables after false-discovery-rate correction. We do not claim positive evidence of equivalence: with a kinematic sub-sample of n = 43, the minimum detectable effect was large, and two one-sided tests (TOST) could not establish equivalence within a meaningful margin (equivalence was not established for any of the six analyzed muscle groups; **Supplementary Table 6**). The null is therefore best interpreted as an absence of between-group differences detectable at this sample size and with this two-dimensional, single-camera, sagittal-plane pipeline, rather than as evidence of biological conservation. The pattern was nonetheless internally consistent: the seven analyzed muscle groups all showed the same null pattern, including the hamstrings, the very muscle group on which the literature places the greatest mechanical demands. The kinematic input itself was rich enough to detect the spatiotemporal differences, so the null finding is not a downstream consequence of underpowered kinematic reconstruction. We interpret these results as indicating that, when matched on the same gait cycle structure, lower-limb muscles showed no detectable between-group differences in length and velocity envelopes, and that performance differences must be sought in dimensions our pipeline did not measure principally muscle force, which requires inverse dynamics and ground reaction force data not available in the current study.

### 4.4 Physiological implications and methodological reflections

Taken together, these findings sketch a coherent picture of what differentiates faster from slower youth male sprinters at the level of neuromuscular function and *in vivo* sprint mechanics. Faster sprinters possess greater hip flexor and hamstring strength qualities that survive age adjustment and therefore reflect trainable neuromuscular capacity rather than developmental status alone, and they translate this strength into shorter ground contact times and longer step lengths during maximum-velocity running, without altering the underlying joint or muscle–tendon kinematic patterns. The acceleration phase, where these strength advantages presumably translate most directly into horizontal impulse production, contributes the largest single share (40.8%) of the total time difference between fastest and slowest performers.

Notably, hamstring eccentric strength and hip flexion isometric strength were independent of chronological age while IMTP and knee extension strength were not. One speculative interpretation is that hamstring eccentric capacity and hip flexion peak force are more sensitive to specific sprint training adaptations such as neural drive, motor unit recruitment and type II fiber contribution, whereas IMTP and knee extension strength are more closely tied to general muscle-mass accretion that accompanies puberty. If correct, this would suggest that hamstring eccentric and hip flexion strength serve as relatively “training-pure” markers of sprint-specific neuromuscular development. Verification would require longitudinal data tracking these qualities through the period of peak height velocity, a clear direction for future work.

Methodologically, the convergence of NordBord, ForceFrame, and standing long jump on the same group of “faster” athletes, combined with the failure of the impulse-based NordBord variables (50–250 ms) to discriminate, suggests that peak and mean force capacities are more diagnostically useful than rate-of-force-development metrics in this population. From a practical standpoint, the comparable cross-validated performance of the laboratory-based predictors and the reduced field-based model (cross-validated R^2^ ≈ 0.40 in both cases) indicates that field-based assessment using standing long jump and Nordic hamstring testing is sufficient for monitoring purposes when laboratory force platforms are unavailable.

### 4.5 Limitations and future directions

Several limitations should be considered. First, the cross-sectional design prevents causal inference about whether neuromuscular strength differences cause or merely accompany sprint performance differences. Second, only male athletes were studied, and the findings may not generalize to female sprinters. Third, biological maturation was estimated only indirectly. A maturity offset was derived from the Mirwald equation using a sitting height calculated as standing height minus measured lower-limb length, rather than a directly measured sitting height, so the maturity estimates carry additional uncertainty. The faster group was significantly more biologically mature than the slower group, and although the principal strength–performance associations were robust to adjustment for this estimated maturity, residual confounding by maturation cannot be fully excluded; longitudinal designs with directly measured maturity indicators are needed to fully disentangle trained capacity from developmental status. Fourth, our musculoskeletal modeling pipeline relied on 2D sagittal-plane kinematics and did not include ground reaction force measurement, ruling out inverse dynamics, static optimization, and direct estimation of muscle forces. The null finding for muscle–tendon kinematics should therefore not be interpreted as evidence that muscle forces are equivalent across performance levels, only that no between-group differences in the geometric (length and velocity) behavior of these muscles were detected. Fifth, the 2D pose estimation approach, while validated for sagittal-plane gait variables, cannot capture frontal-plane or transverse-plane motion, in particular hip rotation and ab/adduction, which contribute to hamstring muscle–tendon length, so any performance-relevant differences in these planes, including those affecting hamstring muscle–tendon excursion, would have been missed.

A further set of limitations concerns the specific markerless motion-capture and modeling pipeline (Sports2D/Pose2Sim). The keypoint confidence thresholds (keypoint likelihood 0.3; average likelihood 0.5) were left at Sports2D defaults rather than tuned to our recording conditions. Touchdown and toe-off events were not detected automatically from foot-marker velocity or vertical position; instead, an operator identified them by frame-by-frame review of the automatically tracked kinematics, which introduces a degree of subjectivity, mitigated, however, by the operator being blind to participants’ group and performance, the athletes and their sprint times being unknown to the marker. The processing pipeline retained only inverse-kinematics joint coordinates; inverse-kinematics fit quality was subsequently quantified as the marker residuals between the scaled model and the tracked sagittal-plane keypoints. Pooled across 43 participants and 170 maximum-velocity trials within the TD1–TO3 analysis window, the mean root-mean-square (RMS) marker error was 28.1 mm (median 24.2 mm; 95th percentile 54.8 mm), the mean maximum marker error was 62.8 mm, and the largest single-frame maximum error was 481.7 mm, with the largest residuals occurring predominantly at the lowest-weighted, most distal head marker. These residuals exceed the thresholds typical of three-dimensional marker-based inverse kinematics (RMS < 20 mm) and should be read as a fit-consistency indicator for the two-dimensional keypoint-derived virtual markers in a single-camera sagittal-plane configuration rather than as a claim of three-dimensional accuracy. Model scaling also followed two non-identical routes—a segment-wise anthropometric scaling used for batch processing (reference model 170 cm/83.81 kg) and the height-based scaling internal to Pose2Sim used for interactive inverse kinematics, which may introduce systematic differences in geometry-derived quantities such as muscle–tendon length, further reinforcing that the muscle–tendon kinematic null should not be over-interpreted. Finally, the pose model was the body_with_feet (HALPE-26-based) configuration, of which 23 keypoints were used, so the analysis was bounded by the anatomical coverage of that keypoint set.

Future studies should integrate ground reaction force measurement (e.g., instrumented force plates or in-shoe pressure sensors) with markerless motion capture to permit full inverse dynamics and muscle force estimation. Longitudinal designs tracking the same athletes through puberty would clarify whether hamstring eccentric and hip flexion strength remain age-independent over time. Finally, training intervention studies could test whether targeted hamstring eccentric and hip flexor strengthening produces commensurate improvements in 50-m sprint performance, providing the causal counterpart to the cross-sectional associations established here.

### 4.6 Conclusion

In youth male sprinters, hip flexor isometric strength and hamstring eccentric strength are the primary neuromuscular discriminators of 50-m sprint performance, independent of chronological age. Standing long jump serves as the strongest single field-based correlate. Poorer hamstring extensibility indexed by a larger popliteal angle showed an exploratory association with slower sprint times that did not survive age adjustment and should be interpreted cautiously pending confirmation. During maximum-velocity running, performance-level differences were detected only in spatiotemporal gait variables (ground contact time, step length, and center-of-mass velocity) in the present analysis; none of the 129 kinematic or 2,480 modeled muscle–tendon kinematic parameters discriminated the groups. These findings are consistent with the view that youth sprint performance is determined principally by the capacity to apply rapid horizontal force during ground contact, and they support training prescriptions that prioritize hamstring eccentric and hip flexor strength development.

## Data Availability

The original contributions presented in the study are included in the article/**Supplementary Material**. Further inquiries can be directed to the corresponding author.
