# Supplementary material for: Neuromuscular strength, not sagittal-plane joint or modeled muscle–tendon kinematics, is associated with 50-m sprint performance in youth male sprinters
Source: Front Physiol. 2026 Jul 22;17:1858508. doi: 10.3389/fphys.2026.1858508 (PMC13439611; doi:10.3389/fphys.2026.1858508)
Supplement: Supplementary file 1 [file Table1.docx]

**Supplementary Materials**

**Supplementary Table S1. Full joint flexibility results across performance groups.**

| **Flexibility measure** | **Faster (n=16)** | **Intermediate (n=15)** | **Slower (n=15)** | **Test** | **F / H** | **p** | **Effect size** | **Post-hoc (F/I, F/S, I/S)** | **FDR p** |
| --- | --- | --- | --- | --- | --- | --- | --- | --- | --- |
| Active hip flexion ROM (°) | 85.28 ± 11.21 | 82.70 ± 9.05 | 92.70 ± 7.75 | ANOVA | 4.50 | 0.017 | 0.173 | ns, ns, ** | 0.052 |
| Passive hip flexion ROM (°) | 110.06 ± 8.05 | 105.27 ± 9.67 | 115.87 ± 11.99 | ANOVA | 4.23 | 0.021 | 0.164 | ns, ns, * | 0.052 |
| Active knee extension ROM / AKE (°) | 159.22 ± 10.05 | 162.40 ± 10.95 | 166.00 ± 13.94 | K-W | 7.55 | 0.023 | 0.129 | ns, *, ns | 0.052 |
| Passive knee extension ROM / PKE (°) | 169.00 ± 20.22 | 175.77 ± 2.58 | 177.30 ± 2.51 | K-W | 4.75 | 0.093 | 0.064 | —, —, — | 0.167 |
| Active hip extension ROM (°) | 30.62 ± 5.42 | 30.17 ± 7.03 | 30.83 ± 7.01 | ANOVA | 0.04 | 0.960 | 0.002 | —, —, — | 0.960 |
| Passive hip extension ROM (°) | 39.81 ± 4.25 | 39.27 ± 5.35 | 40.03 ± 6.41 | ANOVA | 0.08 | 0.923 | 0.004 | —, —, — | 0.960 |
| Active knee flexion ROM (°) | 46.94 ± 4.12 | 47.40 ± 7.81 | 49.17 ± 6.92 | K-W | 1.34 | 0.511 | ≈0 | —, —, — | 0.766 |
| Passive knee flexion ROM (°) | 28.50 ± 3.42 | 28.27 ± 7.14 | 29.03 ± 5.60 | ANOVA | 0.08 | 0.928 | 0.003 | —, —, — | 0.960 |
| Popliteal angle (°) | 38.31 ± 4.31 | 42.20 ± 6.62 | 44.87 ± 3.77 | ANOVA | 6.65 | 0.003 | 0.236 | ns, ***, ns | 0.027 |

*Values are mean ± SD. Test: one-way ANOVA for normally distributed variables, Kruskal–Wallis (K-W) for non-normally distributed variables. Effect size reported as η² for ANOVA and ε² for Kruskal–Wallis. Post-hoc: Tukey HSD or Dunn's test with Bonferroni correction. ns = not significant; * p < 0.05, ** p < 0.01, *** p < 0.001. FDR p-values were computed across the nine flexibility measures using the Benjamini–Hochberg procedure. Negative ε² values (which arise from small-sample variance of the Kruskal–Wallis estimator) are shown as ≈0. All angles are in degrees and are reported as the mean of the left and right limbs; active measures reflect subject-generated motion and passive measures examiner-applied motion to the first firm end-feel. The popliteal angle was obtained in the 90/90 position (hip flexed to 90°) and expresses the residual knee-extension deficit from full extension, so that larger popliteal angles indicate poorer hamstring extensibility (Brosseau et al., 2001). Active knee-extension ROM (AKE) and the popliteal angle both index posterior-chain extensibility but are not interchangeable: AKE is the achieved knee-extension range, whereas the popliteal angle is the residual extension deficit at the standardized 90/90 hip position.*

**Supplementary Table S2. Spearman rank correlations between candidate functional/kinematic variables and 50-m sprint total time.**

| **Variable** | **Category** | **ρ** | **p** | **n** | **FDR p** | **Sig.** |
| --- | --- | --- | --- | --- | --- | --- |
| COM mean velocity (segment) | Kinematics | -0.939 | <0.001 | 42 | <0.001 | * |
| COM peak velocity (segment) | Kinematics | -0.859 | <0.001 | 42 | <0.001 | * |
| Contact time (step 2) | Kinematics | 0.716 | <0.001 | 43 | <0.001 | * |
| Contact time (step 1) | Kinematics | 0.695 | <0.001 | 43 | <0.001 | * |
| Contact time (step 3) | Kinematics | 0.676 | <0.001 | 43 | <0.001 | * |
| FF left hip flexion peak (N) | ForceFrame (isometric) | -0.667 | <0.001 | 44 | <0.001 | * |
| NB left peak impulse (N·s) | NordBord (hamstring eccentric) | -0.655 | <0.001 | 46 | <0.001 | * |
| NB left peak force (N) | NordBord (hamstring eccentric) | -0.644 | <0.001 | 46 | <0.001 | * |
| NB left mean force (N) | NordBord (hamstring eccentric) | -0.640 | <0.001 | 46 | <0.001 | * |
| FF bilateral hip flexion peak (N) | ForceFrame (isometric) | -0.638 | <0.001 | 44 | <0.001 | * |
| NordBord bilateral mean force (N) | NordBord (hamstring eccentric) | -0.637 | <0.001 | 46 | <0.001 | * |
| NordBord bilateral peak force (N) | NordBord (hamstring eccentric) | -0.610 | <0.001 | 46 | <0.001 | * |
| NB right mean force (N) | NordBord (hamstring eccentric) | -0.581 | <0.001 | 46 | <0.001 | * |
| FF right hip flexion peak (N) | ForceFrame (isometric) | -0.574 | <0.001 | 44 | <0.001 | * |
| Contact:flight ratio (step 1) | Kinematics | 0.566 | <0.001 | 43 | <0.001 | * |
| NB right peak force (N) | NordBord (hamstring eccentric) | -0.562 | <0.001 | 46 | <0.001 | * |
| Step length (step 1) | Kinematics | -0.556 | <0.001 | 42 | 0.002 | * |
| FF hip flexion peak / body mass (N/kg) | ForceFrame (isometric) | -0.550 | <0.001 | 43 | 0.002 | * |
| Step length (step 3) | Kinematics | -0.536 | <0.001 | 42 | 0.003 | * |
| Step frequency (step 2, Hz) | Kinematics | -0.531 | <0.001 | 43 | 0.003 | * |
| NB right peak impulse (N·s) | NordBord (hamstring eccentric) | -0.521 | <0.001 | 46 | 0.002 | * |
| Step frequency (step 1, Hz) | Kinematics | -0.490 | <0.001 | 43 | 0.007 | * |
| Popliteal angle (°) | Joint flexibility | 0.487 | <0.001 | 46 | 0.006 | * |
| FF bilateral knee extension peak (N) | ForceFrame (isometric) | -0.486 | <0.001 | 44 | 0.007 | * |
| FF right knee extension peak (N) | ForceFrame (isometric) | -0.481 | <0.001 | 44 | 0.008 | * |
| FF left knee extension peak (N) | ForceFrame (isometric) | -0.475 | 0.001 | 44 | 0.009 | * |
| Step length (step 4) | Kinematics | -0.472 | 0.002 | 42 | 0.012 | * |
| NB left peak force / BM (N/kg) | NordBord (hamstring eccentric) | -0.461 | 0.001 | 45 | 0.011 | * |
| NB left mean force / BM (N/kg) | NordBord (hamstring eccentric) | -0.453 | 0.002 | 45 | 0.013 | * |
| NB left mean torque / BM (Nm/kg) | NordBord (hamstring eccentric) | -0.439 | 0.004 | 41 | 0.026 | * |
| NB left peak torque (Nm) | NordBord (hamstring eccentric) | -0.424 | 0.003 | 46 | 0.023 | * |
| NordBord peak force / body mass (N/kg) | NordBord (hamstring eccentric) | -0.421 | 0.004 | 45 | 0.026 | * |
| Active knee extension ROM (°) | Joint flexibility | 0.416 | 0.004 | 46 | 0.026 | * |
| NB right peak force / BM (N/kg) | NordBord (hamstring eccentric) | -0.415 | 0.005 | 45 | 0.028 | * |
| NB right mean force / BM (N/kg) | NordBord (hamstring eccentric) | -0.402 | 0.006 | 45 | 0.037 | * |
| Trunk angle toe-off 3 | Kinematics | -0.401 | 0.008 | 42 | 0.047 | * |
| Step length (step 2) | Kinematics | -0.401 | 0.009 | 42 | 0.047 | * |
| Passive knee extension ROM (°) | Joint flexibility | 0.385 | 0.008 | 46 | 0.047 | * |
| Trunk angle touchdown 3 | Kinematics | -0.385 | 0.012 | 42 | 0.062 |  |
| FF left knee flexion peak (N) | ForceFrame (isometric) | -0.374 | 0.011 | 45 | 0.060 |  |
| NB right peak torque (Nm) | NordBord (hamstring eccentric) | -0.366 | 0.012 | 46 | 0.063 |  |
| COM vertical oscillation (step 2) | Kinematics | 0.361 | 0.019 | 42 | 0.090 |  |
| FF knee extension peak / body mass (N/kg) | ForceFrame (isometric) | -0.360 | 0.018 | 43 | 0.086 |  |
| NB impulse imbalance (%) | NordBord (hamstring eccentric) | 0.359 | 0.014 | 46 | 0.071 |  |
| Rthigh rom touchdown 1 touchdown 3 | Kinematics | -0.357 | 0.020 | 42 | 0.095 |  |
| NB right mean torque / BM (Nm/kg) | NordBord (hamstring eccentric) | -0.346 | 0.027 | 41 | 0.121 |  |
| FF bilateral hip extension peak (N) | ForceFrame (isometric) | -0.333 | 0.031 | 42 | 0.134 |  |
| Swing thigh rom | Kinematics | -0.330 | 0.033 | 42 | 0.136 |  |
| NB left peak torque / BM (Nm/kg) | NordBord (hamstring eccentric) | -0.329 | 0.028 | 45 | 0.122 |  |
| FF knee flexion peak / body mass (N/kg) | ForceFrame (isometric) | -0.322 | 0.033 | 44 | 0.136 |  |
| FF right hip extension peak (N) | ForceFrame (isometric) | -0.321 | 0.038 | 42 | 0.151 |  |
| Active hip flexion ROM (°) | Joint flexibility | 0.320 | 0.030 | 46 | 0.131 |  |
| NB left mean time to peak (s) | NordBord (hamstring eccentric) | -0.306 | 0.038 | 46 | 0.151 |  |
| FF bilateral knee flexion peak (N) | ForceFrame (isometric) | -0.304 | 0.042 | 45 | 0.164 |  |
| NB left min time to peak (s) | NordBord (hamstring eccentric) | -0.290 | 0.051 | 46 | 0.193 |  |
| FF right knee flexion peak (N) | ForceFrame (isometric) | -0.288 | 0.055 | 45 | 0.205 |  |
| Trunk angle touchdown 2 | Kinematics | -0.288 | 0.064 | 42 | 0.236 |  |
| FF hip flexion:extension ratio | ForceFrame (isometric) | -0.282 | 0.074 | 41 | 0.261 |  |
| NB right mean time to peak (s) | NordBord (hamstring eccentric) | -0.272 | 0.068 | 46 | 0.245 |  |
| NB right peak torque / BM (Nm/kg) | NordBord (hamstring eccentric) | -0.267 | 0.077 | 45 | 0.267 |  |
| Support foot vy touchdown 2 | Kinematics | 0.255 | 0.103 | 42 | 0.342 |  |
| NB left peak impulse at 100 ms (N·s) | NordBord (hamstring eccentric) | -0.252 | 0.091 | 46 | 0.313 |  |
| NordBord time to peak force (s) | NordBord (hamstring eccentric) | -0.247 | 0.098 | 46 | 0.332 |  |
| Rknee min toe-off 1 touchdown 2 | Kinematics | 0.243 | 0.121 | 42 | 0.395 |  |
| FF hip extension peak / body mass (N/kg) | ForceFrame (isometric) | -0.239 | 0.133 | 41 | 0.428 |  |
| Support knee min swing | Kinematics | 0.231 | 0.142 | 42 | 0.449 |  |
| Trunk angle toe-off 1 | Kinematics | -0.222 | 0.159 | 42 | 0.495 |  |
| Lthigh rom touchdown 1 touchdown 3 | Kinematics | -0.214 | 0.174 | 42 | 0.522 |  |
| COM vertical oscillation (step 1) | Kinematics | 0.210 | 0.181 | 42 | 0.533 |  |
| Support thigh rom | Kinematics | -0.209 | 0.184 | 42 | 0.535 |  |
| NB left mean impulse at 200 ms (N·s) | NordBord (hamstring eccentric) | 0.206 | 0.170 | 46 | 0.522 |  |
| NB left mean impulse at 150 ms (N·s) | NordBord (hamstring eccentric) | 0.203 | 0.175 | 46 | 0.522 |  |
| FF left hip extension peak (N) | ForceFrame (isometric) | -0.201 | 0.201 | 42 | 0.569 |  |
| NB right min time to peak (s) | NordBord (hamstring eccentric) | -0.192 | 0.201 | 46 | 0.569 |  |
| Support hip ext rate | Kinematics | -0.187 | 0.235 | 42 | 0.640 |  |
| Lknee min toe-off 2 touchdown 3 | Kinematics | 0.186 | 0.239 | 42 | 0.640 |  |
| NB left mean impulse at 250 ms (N·s) | NordBord (hamstring eccentric) | 0.184 | 0.221 | 46 | 0.616 |  |
| Thigh plus trunk toe-off 3 right | Kinematics | -0.182 | 0.247 | 42 | 0.642 |  |
| Trunk angle touchdown 1 | Kinematics | -0.182 | 0.249 | 42 | 0.642 |  |
| NordBord impulse at 250 ms (N·s) | NordBord (hamstring eccentric) | -0.180 | 0.236 | 45 | 0.640 |  |
| NordBord impulse at 100 ms (N·s) | NordBord (hamstring eccentric) | -0.176 | 0.248 | 45 | 0.642 |  |
| NB left peak impulse at 250 ms (N·s) | NordBord (hamstring eccentric) | -0.172 | 0.254 | 46 | 0.648 |  |
| Passive hip flexion ROM (°) | Joint flexibility | 0.166 | 0.272 | 46 | 0.684 |  |
| Shank vs thigh toe-off 3 left | Kinematics | 0.165 | 0.297 | 42 | 0.730 |  |
| Shank vs thigh toe-off 2 left | Kinematics | 0.163 | 0.304 | 42 | 0.739 |  |
| Trunk angle toe-off 2 | Kinematics | -0.160 | 0.312 | 42 | 0.750 |  |
| NB left mean impulse at 100 ms (N·s) | NordBord (hamstring eccentric) | 0.159 | 0.290 | 46 | 0.722 |  |
| Rthigh rate toe-off 2 touchdown 3 | Kinematics | -0.157 | 0.322 | 42 | 0.755 |  |
| Swing knee min swing | Kinematics | 0.156 | 0.325 | 42 | 0.755 |  |
| NB right peak impulse at 50 ms (N·s) | NordBord (hamstring eccentric) | -0.149 | 0.323 | 46 | 0.755 |  |
| NordBord impulse at 200 ms (N·s) | NordBord (hamstring eccentric) | -0.148 | 0.332 | 45 | 0.758 |  |
| NB left peak impulse at 50 ms (N·s) | NordBord (hamstring eccentric) | -0.147 | 0.334 | 45 | 0.758 |  |
| Swing knee angle toe-off | Kinematics | 0.147 | 0.354 | 42 | 0.796 |  |
| Flight time (step 2) | Kinematics | 0.143 | 0.360 | 43 | 0.799 |  |
| NB right peak impulse at 100 ms (N·s) | NordBord (hamstring eccentric) | -0.134 | 0.376 | 46 | 0.827 |  |
| NB right mean impulse at 150 ms (N·s) | NordBord (hamstring eccentric) | 0.128 | 0.395 | 46 | 0.861 |  |
| Support knee angle touchdown 2 | Kinematics | 0.127 | 0.423 | 42 | 0.900 |  |
| Support ankle angle touchdown | Kinematics | 0.124 | 0.432 | 42 | 0.904 |  |
| NB right mean impulse at 250 ms (N·s) | NordBord (hamstring eccentric) | 0.121 | 0.423 | 46 | 0.900 |  |
| NordBord force asymmetry (%) | NordBord (hamstring eccentric) | 0.120 | 0.427 | 46 | 0.900 |  |
| Lfoot vy touchdown 2 | Kinematics | 0.120 | 0.449 | 42 | 0.919 |  |
| NordBord impulse at 50 ms (N·s) | NordBord (hamstring eccentric) | -0.119 | 0.441 | 44 | 0.913 |  |
| Swing hip ext rate | Kinematics | 0.116 | 0.464 | 42 | 0.942 |  |
| Lthigh rate toe-off 2 touchdown 3 | Kinematics | 0.114 | 0.474 | 42 | 0.943 |  |
| NB right mean impulse at 200 ms (N·s) | NordBord (hamstring eccentric) | 0.109 | 0.471 | 46 | 0.943 |  |
| Swing thigh minus shank min touchdown 2 toe-off 2 left | Kinematics | 0.107 | 0.499 | 42 | 0.985 |  |
| Support com dx touchdown 2 left | Kinematics | -0.102 | 0.522 | 42 | 0.993 |  |
| Lknee min toe-off 1 touchdown 2 | Kinematics | 0.100 | 0.527 | 42 | 0.993 |  |
| Rfoot vy touchdown 2 | Kinematics | 0.099 | 0.531 | 42 | 0.993 |  |
| NB max force imbalance (%) | NordBord (hamstring eccentric) | 0.094 | 0.535 | 46 | 0.993 |  |
| Support com dx touchdown 3 right | Kinematics | -0.094 | 0.555 | 42 | 0.993 |  |
| Swing foot vy touchdown | Kinematics | 0.093 | 0.556 | 42 | 0.993 |  |
| NB mean force imbalance (%) | NordBord (hamstring eccentric) | 0.092 | 0.544 | 46 | 0.993 |  |
| Toeoff com dx toe-off 1 right | Kinematics | -0.089 | 0.575 | 42 | 0.993 |  |
| Thigh plus trunk touchdown 3 right | Kinematics | -0.089 | 0.576 | 42 | 0.993 |  |
| NB left peak impulse at 200 ms (N·s) | NordBord (hamstring eccentric) | -0.085 | 0.574 | 46 | 0.993 |  |
| NordBord impulse at 150 ms (N·s) | NordBord (hamstring eccentric) | -0.085 | 0.579 | 45 | 0.993 |  |
| FF hamstring:quadriceps ratio | ForceFrame (isometric) | 0.083 | 0.593 | 44 | 0.993 |  |
| Swing thigh minus shank min touchdown 3 toe-off 3 left | Kinematics | 0.081 | 0.609 | 42 | 0.993 |  |
| Thigh plus trunk touchdown 1 left | Kinematics | 0.078 | 0.625 | 42 | 0.993 |  |
| Swing hip angle touchdown 2 | Kinematics | -0.077 | 0.629 | 42 | 0.993 |  |
| Rknee angle touchdown 2 | Kinematics | 0.073 | 0.644 | 42 | 0.993 |  |
| Rthigh rate toe-off 1 touchdown 2 | Kinematics | 0.073 | 0.646 | 42 | 0.993 |  |
| Shank vs thigh toe-off 1 left | Kinematics | 0.073 | 0.648 | 42 | 0.993 |  |
| Support hip angle touchdown 2 | Kinematics | 0.071 | 0.656 | 42 | 0.993 |  |
| Rknee angle toe-off 1 | Kinematics | 0.068 | 0.668 | 42 | 0.993 |  |
| Thigh plus trunk toe-off 1 right | Kinematics | -0.068 | 0.669 | 42 | 0.993 |  |
| Rknee min touchdown 1 toe-off 1 | Kinematics | 0.065 | 0.683 | 42 | 0.993 |  |
| Rfoot vy touchdown 1 | Kinematics | 0.065 | 0.685 | 42 | 0.993 |  |
| Swing thigh minus shank min touchdown 1 toe-off 1 right | Kinematics | 0.064 | 0.687 | 42 | 0.993 |  |
| Lknee angle touchdown 2 | Kinematics | 0.063 | 0.692 | 42 | 0.993 |  |
| Swing foot vy touchdown 2 | Kinematics | -0.063 | 0.693 | 42 | 0.993 |  |
| Swing knee min stance | Kinematics | 0.063 | 0.693 | 42 | 0.993 |  |
| Lthigh rate toe-off 1 touchdown 2 | Kinematics | -0.063 | 0.694 | 42 | 0.993 |  |
| NB left mean impulse at 50 ms (N·s) | NordBord (hamstring eccentric) | -0.062 | 0.682 | 46 | 0.993 |  |
| Support knee angle touchdown | Kinematics | 0.061 | 0.703 | 42 | 0.993 |  |
| Flight time (step 1) | Kinematics | -0.060 | 0.700 | 43 | 0.993 |  |
| Knee sep touchdown 2 | Kinematics | 0.060 | 0.704 | 42 | 0.993 |  |
| Lhip angle touchdown 2 | Kinematics | -0.060 | 0.707 | 42 | 0.993 |  |
| Toeoff com dx toe-off 2 left | Kinematics | -0.059 | 0.712 | 42 | 0.993 |  |
| Shank vs thigh toe-off 3 right | Kinematics | 0.058 | 0.713 | 42 | 0.993 |  |
| Rknee angle touchdown 1 | Kinematics | 0.057 | 0.722 | 42 | 0.993 |  |
| Toeoff com dx toe-off 1 left | Kinematics | -0.056 | 0.724 | 42 | 0.993 |  |
| Thigh plus trunk touchdown 1 right | Kinematics | -0.055 | 0.728 | 42 | 0.993 |  |
| Lthigh rate touchdown 1 toe-off 1 | Kinematics | -0.055 | 0.729 | 42 | 0.993 |  |
| Passive hip extension ROM (°) | Joint flexibility | 0.051 | 0.734 | 46 | 0.993 |  |
| Thigh plus trunk touchdown 2 left | Kinematics | -0.050 | 0.754 | 42 | 0.993 |  |
| Swing ankle angle touchdown | Kinematics | -0.050 | 0.755 | 42 | 0.993 |  |
| Rknee min toe-off 2 touchdown 3 | Kinematics | 0.050 | 0.755 | 42 | 0.993 |  |
| Support foot vy touchdown | Kinematics | -0.048 | 0.765 | 42 | 0.993 |  |
| Swing knee angle touchdown 2 | Kinematics | 0.046 | 0.771 | 42 | 0.993 |  |
| Rshank rate toe-off 2 touchdown 3 | Kinematics | -0.046 | 0.773 | 42 | 0.993 |  |
| Thigh plus trunk touchdown 3 left | Kinematics | -0.046 | 0.774 | 42 | 0.993 |  |
| Active hip extension ROM (°) | Joint flexibility | -0.042 | 0.782 | 46 | 0.993 |  |
| Swing thigh minus shank min touchdown 2 toe-off 2 right | Kinematics | -0.042 | 0.793 | 42 | 0.993 |  |
| Swing knee angle touchdown | Kinematics | 0.039 | 0.808 | 42 | 0.993 |  |
| Toeoff com dx toe-off 2 right | Kinematics | -0.038 | 0.813 | 42 | 0.993 |  |
| Shank vs thigh toe-off 1 right | Kinematics | 0.037 | 0.817 | 42 | 0.993 |  |
| Support com dx touchdown 1 right | Kinematics | -0.037 | 0.818 | 42 | 0.993 |  |
| Shank vs thigh toe-off 2 right | Kinematics | -0.036 | 0.819 | 42 | 0.993 |  |
| Lankle angle touchdown 1 | Kinematics | 0.036 | 0.822 | 42 | 0.993 |  |
| Thigh plus trunk toe-off 2 right | Kinematics | -0.036 | 0.823 | 42 | 0.993 |  |
| Knee sep touchdown 3 | Kinematics | -0.035 | 0.825 | 42 | 0.993 |  |
| Passive knee flexion ROM (°) | Joint flexibility | 0.033 | 0.828 | 46 | 0.993 |  |
| NB right mean impulse at 100 ms (N·s) | NordBord (hamstring eccentric) | 0.033 | 0.828 | 46 | 0.993 |  |
| Swing thigh plus trunk min ft2 left | Kinematics | 0.033 | 0.838 | 42 | 0.993 |  |
| Support hip angle toe-off | Kinematics | -0.032 | 0.842 | 42 | 0.993 |  |
| Support com dx touchdown 3 left | Kinematics | -0.030 | 0.849 | 42 | 0.993 |  |
| Support knee angle toe-off | Kinematics | -0.030 | 0.851 | 42 | 0.993 |  |
| Knee sep touchdown 1 | Kinematics | -0.029 | 0.856 | 42 | 0.993 |  |
| Lshank rate toe-off 1 touchdown 2 | Kinematics | -0.029 | 0.858 | 42 | 0.993 |  |
| Rthigh rate touchdown 1 toe-off 1 | Kinematics | 0.028 | 0.862 | 42 | 0.993 |  |
| Lshank rate toe-off 2 touchdown 3 | Kinematics | 0.028 | 0.862 | 42 | 0.993 |  |
| Rhip angle touchdown 2 | Kinematics | 0.028 | 0.862 | 42 | 0.993 |  |
| Swing hip angle toe-off | Kinematics | -0.024 | 0.879 | 42 | 0.993 |  |
| Swing thigh plus trunk min ft1 left | Kinematics | 0.024 | 0.880 | 42 | 0.993 |  |
| Swing hip angle touchdown | Kinematics | -0.022 | 0.889 | 42 | 0.993 |  |
| Lhip ext rate touchdown 1 toe-off 1 | Kinematics | -0.021 | 0.893 | 42 | 0.993 |  |
| Swing thigh plus trunk min ft2 right | Kinematics | -0.021 | 0.896 | 42 | 0.993 |  |
| Support com dx touchdown 2 right | Kinematics | -0.020 | 0.899 | 42 | 0.993 |  |
| NB right mean impulse at 50 ms (N·s) | NordBord (hamstring eccentric) | 0.020 | 0.895 | 46 | 0.993 |  |
| Active knee flexion ROM (°) | Joint flexibility | 0.020 | 0.895 | 46 | 0.993 |  |
| Rhip angle touchdown 1 | Kinematics | -0.020 | 0.902 | 42 | 0.993 |  |
| Rankle angle touchdown 1 | Kinematics | -0.018 | 0.910 | 42 | 0.993 |  |
| NB left peak impulse at 150 ms (N·s) | NordBord (hamstring eccentric) | -0.016 | 0.917 | 46 | 0.993 |  |
| Swing thigh minus shank min touchdown 1 toe-off 1 left | Kinematics | 0.016 | 0.922 | 42 | 0.993 |  |
| Lfoot vy touchdown 1 | Kinematics | 0.015 | 0.924 | 42 | 0.993 |  |
| Lhip angle toe-off 1 | Kinematics | -0.013 | 0.937 | 42 | 0.993 |  |
| Support knee min stance | Kinematics | 0.013 | 0.937 | 42 | 0.993 |  |
| Rthigh rate touchdown 2 toe-off 2 | Kinematics | -0.012 | 0.941 | 42 | 0.993 |  |
| Swing thigh minus shank min touchdown 3 toe-off 3 right | Kinematics | -0.011 | 0.946 | 42 | 0.993 |  |
| Support com dx touchdown 1 left | Kinematics | 0.011 | 0.947 | 42 | 0.993 |  |
| Thigh plus trunk touchdown 2 right | Kinematics | -0.011 | 0.948 | 42 | 0.993 |  |
| Rhip angle toe-off 1 | Kinematics | 0.009 | 0.956 | 42 | 0.993 |  |
| Thigh plus trunk toe-off 2 left | Kinematics | -0.009 | 0.957 | 42 | 0.993 |  |
| Lknee min touchdown 1 toe-off 1 | Kinematics | 0.008 | 0.959 | 42 | 0.993 |  |
| Swing thigh plus trunk min ft1 right | Kinematics | -0.008 | 0.960 | 42 | 0.993 |  |
| Rshank rate toe-off 1 touchdown 2 | Kinematics | -0.007 | 0.963 | 42 | 0.993 |  |
| Rhip ext rate touchdown 1 toe-off 1 | Kinematics | -0.007 | 0.966 | 42 | 0.993 |  |
| Lhip angle touchdown 1 | Kinematics | -0.006 | 0.969 | 42 | 0.993 |  |
| Lthigh rate touchdown 2 toe-off 2 | Kinematics | 0.006 | 0.970 | 42 | 0.993 |  |
| NB right peak impulse at 250 ms (N·s) | NordBord (hamstring eccentric) | 0.006 | 0.970 | 46 | 0.993 |  |
| NB right peak impulse at 150 ms (N·s) | NordBord (hamstring eccentric) | 0.004 | 0.978 | 46 | 0.993 |  |
| Lknee angle touchdown 1 | Kinematics | 0.004 | 0.982 | 42 | 0.993 |  |
| Thigh plus trunk toe-off 1 left | Kinematics | -0.003 | 0.985 | 42 | 0.993 |  |
| NB right peak impulse at 200 ms (N·s) | NordBord (hamstring eccentric) | 0.003 | 0.985 | 46 | 0.993 |  |
| Thigh plus trunk toe-off 3 left | Kinematics | 0.002 | 0.988 | 42 | 0.993 |  |
| Support hip angle touchdown | Kinematics | 0.002 | 0.990 | 42 | 0.993 |  |
| Lknee angle toe-off 1 | Kinematics | -0.001 | 0.993 | 42 | 0.993 |  |

*Spearman's rank correlation coefficient (ρ) between each candidate variable and 50-m total sprint time. Negative ρ indicates that higher values of the variable are associated with faster (lower) sprint times. FDR p-values were computed using the Benjamini–Hochberg procedure across all 209 variables. Variables marked * reached FDR-corrected significance at q < 0.05. Sample size (n) varies because not all participants completed every test. The NordBord block includes all individual per-side and bilateral force, impulse, and rate-of-force-development metrics extracted from the raw NordBord output (53 variables); main manuscript Table 3 presents only the five most representative summary measures.*

**Supplementary Table S3. Spearman correlations between neuromuscular and flexibility variables and 50-m sprint segment times.**

| **Variable** | **n** | **0–20 m** | **20–30 m** | **30–40 m** | **40–50 m** | **0–50 m total** |
| --- | --- | --- | --- | --- | --- | --- |
| NordBord bilateral mean force (N) | 46 | −0.642* | −0.530* | −0.529* | −0.570* | −0.637* |
| NordBord bilateral peak force (N) | 46 | −0.629* | −0.466* | −0.535* | −0.545* | −0.610* |
| NordBord peak force / body mass (N/kg) | 44 | −0.565* | −0.416* | −0.572* | −0.516* | −0.572* |
| NordBord time to peak force (s) | 46 | −0.208 | −0.267 | −0.264 | −0.237 | −0.247 |
| NordBord impulse at 100 ms (N·s) | 46 | −0.263 | −0.147 | −0.140 | −0.165 | −0.223 |
| NordBord impulse at 50 ms (N·s) | 45 | −0.220 | −0.145 | −0.120 | −0.102 | −0.170 |
| NordBord force asymmetry (%) | 46 | +0.086 | +0.209 | +0.160 | +0.049 | +0.120 |
| NordBord impulse at 250 ms (N·s) | 46 | −0.154 | −0.054 | −0.028 | −0.054 | −0.109 |
| NordBord impulse at 200 ms (N·s) | 46 | −0.122 | −0.014 | +0.014 | −0.042 | −0.078 |
| NordBord impulse at 150 ms (N·s) | 46 | −0.069 | +0.048 | +0.066 | +0.012 | −0.018 |
| IMTP peak force (N) | 46 | −0.459* | −0.494* | −0.357* | −0.381* | −0.488* |
| Standing long jump (m) | 46 | −0.688* | −0.661* | −0.458* | −0.607* | −0.684* |
| FF bilateral hip flexion peak (N) | 44 | −0.631* | −0.416* | −0.661* | −0.573* | −0.638* |
| FF hip flexion peak / body mass (N/kg) | 32 | −0.545* | −0.492* | −0.672* | −0.530* | −0.581* |
| FF bilateral knee extension peak (N) | 44 | −0.477* | −0.436* | −0.445* | −0.410* | −0.486* |
| FF knee flexion peak / body mass (N/kg) | 33 | −0.403 | −0.261 | −0.323 | −0.251 | −0.362 |
| FF bilateral hip extension peak (N) | 42 | −0.338 | −0.228 | −0.195 | −0.226 | −0.333 |
| FF bilateral knee flexion peak (N) | 45 | −0.325 | −0.279 | −0.279 | −0.182 | −0.304 |
| FF knee extension peak / body mass (N/kg) | 32 | −0.306 | −0.254 | −0.287 | −0.250 | −0.288 |
| FF hip flexion:extension ratio | 41 | −0.261 | −0.197 | −0.300 | −0.358 | −0.282 |
| FF hip extension peak / body mass (N/kg) | 30 | −0.292 | −0.132 | −0.192 | −0.117 | −0.250 |
| FF hamstring:quadriceps ratio | 44 | +0.062 | +0.039 | +0.174 | +0.130 | +0.083 |
| Popliteal angle (°) | 46 | +0.500* | +0.441* | +0.474* | +0.423* | +0.487* |
| Active knee extension ROM (°) | 46 | +0.441* | +0.449* | +0.356* | +0.328 | +0.416* |
| Passive knee extension ROM (°) | 46 | +0.384* | +0.343 | +0.354* | +0.276 | +0.385* |
| Active hip flexion ROM (°) | 46 | +0.298 | +0.302 | +0.338 | +0.250 | +0.320 |
| Passive hip flexion ROM (°) | 46 | +0.128 | +0.213 | +0.287 | +0.174 | +0.166 |
| Passive hip extension ROM (°) | 46 | +0.006 | +0.023 | +0.041 | +0.125 | +0.051 |
| Active hip extension ROM (°) | 46 | −0.060 | −0.064 | −0.015 | +0.024 | −0.042 |
| Passive knee flexion ROM (°) | 46 | −0.026 | +0.075 | +0.108 | +0.064 | +0.033 |
| Active knee flexion ROM (°) | 46 | −0.012 | +0.087 | +0.139 | +0.020 | +0.020 |

*Spearman's ρ between each candidate neuromuscular/flexibility variable and the five 50-m sprint split intervals. * indicates FDR-corrected significance at q < 0.05 (Benjamini–Hochberg, applied across all 155 IV × DV combinations).*

**Supplementary Table S4. Full kinematic variable results across performance groups with FDR correction.**

| **Variable** | **Type** | **Test** | **Statistic** | **p** | **Effect size** | **Faster (mean ± SD)** | **Intermediate (mean ± SD)** | **Slower (mean ± SD)** | **FDR p** | **Post-hoc** |
| --- | --- | --- | --- | --- | --- | --- | --- | --- | --- | --- |
| COM mean velocity (segment) | Spatiotemporal | K-W | 33.57 | <0.001 | 0.809 | 10.55 ± 0.28 | 9.98 ± 0.23 | 8.99 ± 0.37 | <0.001 | F/I***, F/S***, I/S*** |
| COM peak velocity (segment) | Spatiotemporal | K-W | 30.31 | <0.001 | 0.726 | 10.93 ± 0.30 | 10.45 ± 0.32 | 9.46 ± 0.50 | <0.001 | F/I**, F/S***, I/S*** |
| Contact time (step 2) | Spatiotemporal | ANOVA | 15.67 | <0.001 | 0.439 | 0.10 ± 0.01 | 0.11 ± 0.01 | 0.12 ± 0.01 | <0.001 | F/I*, F/S***, I/S* |
| Contact time (step 1) | Spatiotemporal | ANOVA | 14.70 | <0.001 | 0.424 | 0.10 ± 0.01 | 0.10 ± 0.00 | 0.11 ± 0.01 | <0.001 | F/I*, F/S***, I/S* |
| Contact time (step 3) | Spatiotemporal | K-W | 17.75 | <0.001 | 0.394 | 0.10 ± 0.01 | 0.11 ± 0.01 | 0.12 ± 0.01 | 0.004 | F/S***, I/S** |
| Step length (step 1) | Spatiotemporal | ANOVA | 10.09 | <0.001 | 0.341 | 2.24 ± 0.11 | 2.20 ± 0.11 | 2.05 ± 0.12 | 0.006 | F/S***, I/S** |
| Step length (step 3) | Spatiotemporal | ANOVA | 8.78 | <0.001 | 0.310 | 2.26 ± 0.11 | 2.21 ± 0.12 | 2.09 ± 0.11 | 0.013 | F/S***, I/S* |
| Contact:flight ratio (step 1) | Spatiotemporal | ANOVA | 7.08 | 0.002 | 0.261 | 0.84 ± 0.08 | 0.89 ± 0.08 | 0.98 ± 0.13 | 0.038 | F/S** |
| Step length (step 4) | Spatiotemporal | ANOVA | 6.92 | 0.003 | 0.262 | 2.36 ± 0.13 | 2.31 ± 0.13 | 2.18 ± 0.12 | 0.038 | F/S**, I/S* |
| Step length (step 2) | Spatiotemporal | K-W | 11.14 | 0.004 | 0.234 | 2.37 ± 0.20 | 2.29 ± 0.12 | 2.17 ± 0.11 | 0.049 | F/S**, I/S* |
| Step frequency (step 1, Hz) | Spatiotemporal | ANOVA | 5.26 | 0.009 | 0.208 | 4.64 ± 0.18 | 4.54 ± 0.23 | 4.37 ± 0.25 | 0.110 | F/S** |
| COM vertical oscillation (step 2) | Spatiotemporal | ANOVA | 3.50 | 0.040 | 0.152 | 0.03 ± 0.01 | 0.04 ± 0.01 | 0.04 ± 0.01 | 0.344 | — |
| Step frequency (step 2, Hz) | Spatiotemporal | ANOVA | 3.37 | 0.045 | 0.144 | 4.53 ± 0.47 | 4.39 ± 0.20 | 4.21 ± 0.26 | 0.359 | — |
| COM vertical oscillation (step 1) | Spatiotemporal | ANOVA | 1.08 | 0.348 | 0.053 | 0.03 ± 0.01 | 0.03 ± 0.01 | 0.03 ± 0.01 | 0.811 | — |
| Flight time (step 1) | Spatiotemporal | ANOVA | 0.05 | 0.948 | 0.003 | 0.12 ± 0.01 | 0.12 ± 0.01 | 0.12 ± 0.01 | 0.982 | — |
| Flight time (step 2) | Spatiotemporal | K-W | 0.10 | 0.951 | ≈0 | 0.13 ± 0.03 | 0.12 ± 0.01 | 0.12 ± 0.01 | 0.982 | — |
| Trunk angle toe-off 3 | Joint angle | ANOVA | 4.83 | 0.013 | 0.198 | 100.63 ± 4.26 | 97.95 ± 3.74 | 96.27 ± 3.11 | 0.144 | F/S* |
| Lknee min toe-off 2 touchdown 3 | Joint angle | K-W | 5.09 | 0.078 | 0.079 | 73.92 ± 28.89 | 62.80 ± 10.62 | 79.71 ± 20.59 | 0.573 | — |
| Rknee min toe-off 1 touchdown 2 | Joint angle | K-W | 4.93 | 0.085 | 0.075 | 80.98 ± 28.09 | 71.37 ± 11.39 | 86.95 ± 17.53 | 0.573 | — |
| Trunk angle touchdown 3 | Joint angle | ANOVA | 2.58 | 0.089 | 0.117 | 103.30 ± 3.22 | 101.09 ± 3.99 | 100.43 ± 3.36 | 0.573 | — |
| Trunk angle toe-off 2 | Joint angle | ANOVA | 2.15 | 0.131 | 0.099 | 98.38 ± 3.05 | 95.88 ± 3.77 | 96.93 ± 2.92 | 0.728 | — |
| Trunk angle touchdown 2 | Joint angle | ANOVA | 2.10 | 0.136 | 0.097 | 101.08 ± 2.36 | 99.26 ± 2.56 | 99.45 ± 3.00 | 0.728 | — |
| Lknee min toe-off 1 touchdown 2 | Joint angle | K-W | 3.82 | 0.148 | 0.047 | 70.56 ± 25.58 | 84.39 ± 21.91 | 71.06 ± 15.84 | 0.736 | — |
| Lhip angle touchdown 1 | Joint angle | K-W | 3.11 | 0.211 | 0.028 | 150.53 ± 15.36 | 144.28 ± 13.62 | 150.16 ± 10.26 | 0.811 | — |
| Swing knee angle toe-off | Joint angle | K-W | 2.90 | 0.235 | 0.023 | 95.23 ± 21.35 | 99.37 ± 11.12 | 107.41 ± 11.99 | 0.811 | — |
| Rknee min toe-off 2 touchdown 3 | Joint angle | K-W | 2.86 | 0.240 | 0.022 | 72.29 ± 25.95 | 83.68 ± 21.88 | 72.22 ± 18.74 | 0.811 | — |
| Support knee min swing | Joint angle | K-W | 2.45 | 0.294 | 0.012 | 71.21 ± 30.44 | 74.11 ± 18.72 | 69.69 ± 13.29 | 0.811 | — |
| Lhip angle toe-off 1 | Joint angle | K-W | 2.07 | 0.355 | 0.002 | 141.35 ± 23.12 | 131.11 ± 22.03 | 142.63 ± 19.80 | 0.811 | — |
| Lknee angle touchdown 1 | Joint angle | K-W | 1.70 | 0.427 | ≈0 | 109.99 ± 39.27 | 95.53 ± 33.49 | 113.29 ± 31.27 | 0.874 | — |
| Rknee angle touchdown 2 | Joint angle | K-W | 1.42 | 0.492 | ≈0 | 109.85 ± 38.90 | 99.93 ± 31.69 | 115.92 ± 28.71 | 0.907 | — |
| Lankle angle touchdown 1 | Joint angle | ANOVA | 0.70 | 0.501 | 0.035 | 116.59 ± 10.46 | 119.62 ± 9.18 | 115.37 ± 9.23 | 0.911 | — |
| Lknee angle touchdown 2 | Joint angle | K-W | 1.32 | 0.517 | ≈0 | 100.77 ± 40.70 | 116.07 ± 35.69 | 96.83 ± 32.66 | 0.914 | — |
| Swing knee min swing | Joint angle | ANOVA | 0.63 | 0.541 | 0.031 | 80.33 ± 23.03 | 81.65 ± 17.88 | 88.32 ± 18.17 | 0.921 | — |
| Rhip angle toe-off 1 | Joint angle | ANOVA | 0.62 | 0.544 | 0.031 | 144.18 ± 22.19 | 149.52 ± 20.19 | 140.82 ± 18.94 | 0.921 | — |
| Support knee angle touchdown 2 | Joint angle | K-W | 1.19 | 0.551 | ≈0 | 81.76 ± 33.33 | 86.55 ± 26.03 | 83.28 ± 21.68 | 0.921 | — |
| Trunk angle toe-off 1 | Joint angle | ANOVA | 0.59 | 0.557 | 0.030 | 97.24 ± 2.47 | 96.42 ± 2.53 | 96.26 ± 2.76 | 0.921 | — |
| Trunk angle touchdown 1 | Joint angle | ANOVA | 0.55 | 0.581 | 0.027 | 100.25 ± 2.53 | 99.64 ± 2.44 | 99.18 ± 3.12 | 0.936 | — |
| Support ankle angle touchdown | Joint angle | ANOVA | 0.54 | 0.590 | 0.027 | 109.16 ± 7.55 | 111.62 ± 8.05 | 111.58 ± 6.23 | 0.937 | — |
| Support hip angle touchdown | Joint angle | K-W | 0.87 | 0.646 | ≈0 | 141.58 ± 16.63 | 143.37 ± 8.94 | 145.67 ± 7.55 | 0.937 | — |
| Rknee angle touchdown 1 | Joint angle | K-W | 0.83 | 0.662 | ≈0 | 104.06 ± 36.74 | 116.31 ± 33.95 | 101.02 ± 32.25 | 0.937 | — |
| Rknee min touchdown 1 toe-off 1 | Joint angle | K-W | 0.82 | 0.663 | ≈0 | 93.01 ± 36.32 | 101.98 ± 33.96 | 90.44 ± 31.03 | 0.937 | — |
| Rhip angle touchdown 1 | Joint angle | ANOVA | 0.40 | 0.673 | 0.020 | 150.84 ± 17.77 | 155.07 ± 8.82 | 153.89 ± 10.43 | 0.937 | — |
| Support knee angle toe-off | Joint angle | K-W | 0.79 | 0.675 | ≈0 | 134.12 ± 15.29 | 134.62 ± 17.20 | 130.50 ± 14.60 | 0.937 | — |
| Swing hip angle toe-off | Joint angle | K-W | 0.77 | 0.680 | ≈0 | 129.88 ± 19.33 | 127.27 ± 20.61 | 129.63 ± 15.60 | 0.937 | — |
| Lknee angle toe-off 1 | Joint angle | ANOVA | 0.38 | 0.687 | 0.019 | 112.01 ± 27.27 | 118.84 ± 22.22 | 118.23 ± 19.42 | 0.937 | — |
| Rhip angle touchdown 2 | Joint angle | K-W | 0.74 | 0.689 | ≈0 | 154.39 ± 14.92 | 152.53 ± 13.97 | 154.46 ± 10.90 | 0.937 | — |
| Swing hip angle touchdown | Joint angle | K-W | 0.73 | 0.693 | ≈0 | 159.79 ± 9.88 | 155.98 ± 12.66 | 158.38 ± 8.79 | 0.937 | — |
| Swing knee min stance | Joint angle | K-W | 0.68 | 0.710 | ≈0 | 66.78 ± 21.32 | 70.81 ± 20.16 | 73.22 ± 21.18 | 0.937 | — |
| Lknee min touchdown 1 toe-off 1 | Joint angle | ANOVA | 0.33 | 0.720 | 0.017 | 93.65 ± 35.62 | 88.32 ± 34.32 | 98.83 ± 30.14 | 0.937 | — |
| Lhip angle touchdown 2 | Joint angle | K-W | 0.65 | 0.724 | ≈0 | 144.57 ± 16.68 | 149.59 ± 11.04 | 147.21 ± 12.48 | 0.937 | — |
| Swing ankle angle touchdown | Joint angle | K-W | 0.45 | 0.800 | ≈0 | 128.13 ± 9.39 | 127.55 ± 7.37 | 126.67 ± 9.02 | 0.946 | — |
| Swing hip angle touchdown 2 | Joint angle | K-W | 0.45 | 0.800 | ≈0 | 142.54 ± 17.79 | 144.42 ± 8.04 | 146.25 ± 10.27 | 0.946 | — |
| Rankle angle touchdown 1 | Joint angle | ANOVA | 0.22 | 0.804 | 0.011 | 120.70 ± 14.82 | 119.55 ± 13.09 | 122.88 ± 11.29 | 0.946 | — |
| Rknee angle toe-off 1 | Joint angle | K-W | 0.41 | 0.814 | ≈0 | 117.33 ± 27.22 | 115.15 ± 24.24 | 119.68 ± 16.35 | 0.946 | — |
| Swing knee angle touchdown | Joint angle | K-W | 0.34 | 0.843 | ≈0 | 84.58 ± 32.45 | 84.75 ± 27.13 | 86.39 ± 23.13 | 0.952 | — |
| Support hip angle touchdown 2 | Joint angle | ANOVA | 0.12 | 0.888 | 0.006 | 156.42 ± 11.52 | 157.71 ± 12.72 | 155.43 ± 12.32 | 0.971 | — |
| Support knee min stance | Joint angle | K-W | 0.17 | 0.918 | ≈0 | 119.88 ± 24.96 | 119.49 ± 27.37 | 116.06 ± 21.59 | 0.982 | — |
| Support knee angle touchdown | Joint angle | K-W | 0.07 | 0.967 | ≈0 | 129.47 ± 27.89 | 127.10 ± 28.36 | 127.92 ± 25.10 | 0.982 | — |
| Support hip angle toe-off | Joint angle | K-W | 0.05 | 0.977 | ≈0 | 155.65 ± 17.38 | 153.36 ± 16.85 | 153.82 ± 13.87 | 0.982 | — |
| Swing knee angle touchdown 2 | Joint angle | K-W | 0.04 | 0.982 | ≈0 | 128.87 ± 30.24 | 129.44 ± 27.39 | 129.47 ± 21.49 | 0.982 | — |
| Lthigh rate toe-off 2 touchdown 3 | Angular velocity | ANOVA | 1.85 | 0.171 | 0.087 | 8.05 ± 246.68 | -87.08 ± 246.14 | 83.75 ± 193.55 | 0.759 | — |
| Rthigh rate touchdown 1 toe-off 1 | Angular velocity | K-W | 2.58 | 0.276 | 0.015 | -89.06 ± 485.36 | 106.31 ± 372.72 | -149.46 ± 312.21 | 0.811 | — |
| Rshank rate toe-off 2 touchdown 3 | Angular velocity | K-W | 2.57 | 0.277 | 0.014 | 190.65 ± 426.61 | -1.15 ± 294.50 | 210.05 ± 259.40 | 0.811 | — |
| Lthigh rate toe-off 1 touchdown 2 | Angular velocity | K-W | 2.40 | 0.301 | 0.010 | -8.65 ± 251.87 | 76.85 ± 246.90 | -69.71 ± 210.88 | 0.811 | — |
| Rthigh rate toe-off 1 touchdown 2 | Angular velocity | ANOVA | 1.24 | 0.302 | 0.060 | 45.98 ± 255.34 | -33.26 ± 221.13 | 102.81 ± 193.76 | 0.811 | — |
| Rthigh rate toe-off 2 touchdown 3 | Angular velocity | ANOVA | 1.23 | 0.304 | 0.059 | 27.57 ± 223.76 | 63.79 ± 206.54 | -58.84 ± 189.76 | 0.811 | — |
| Lshank rate toe-off 1 touchdown 2 | Angular velocity | ANOVA | 1.17 | 0.322 | 0.056 | 179.12 ± 414.82 | 32.71 ± 284.96 | 220.16 ± 288.76 | 0.811 | — |
| Rthigh rate touchdown 2 toe-off 2 | Angular velocity | K-W | 2.16 | 0.340 | 0.004 | 10.45 ± 482.12 | -167.47 ± 373.37 | 89.49 ± 338.74 | 0.811 | — |
| Support hip ext rate | Angular velocity | K-W | 2.08 | 0.353 | 0.002 | 140.53 ± 187.94 | 96.69 ± 159.79 | 67.16 ± 138.97 | 0.811 | — |
| Lthigh rate touchdown 1 toe-off 1 | Angular velocity | K-W | 1.65 | 0.438 | ≈0 | 41.34 ± 494.50 | -136.57 ± 387.98 | 117.19 ± 348.71 | 0.880 | — |
| Lthigh rate touchdown 2 toe-off 2 | Angular velocity | K-W | 1.56 | 0.460 | ≈0 | -57.92 ± 509.79 | 130.79 ± 402.52 | -150.21 ± 345.09 | 0.880 | — |
| Lshank rate toe-off 2 touchdown 3 | Angular velocity | ANOVA | 0.79 | 0.460 | 0.039 | 75.73 ± 433.59 | 181.69 ± 295.54 | 19.70 ± 254.66 | 0.880 | — |
| Rshank rate toe-off 1 touchdown 2 | Angular velocity | K-W | 1.23 | 0.541 | ≈0 | 66.23 ± 357.69 | 178.87 ± 296.46 | 33.12 ± 231.57 | 0.921 | — |
| Swing hip ext rate | Angular velocity | ANOVA | 0.29 | 0.747 | 0.015 | -302.66 ± 200.72 | -275.51 ± 203.72 | -248.55 ± 145.21 | 0.945 | — |
| Rhip ext rate touchdown 1 toe-off 1 | Angular velocity | ANOVA | 0.16 | 0.854 | 0.008 | -68.47 ± 303.70 | -57.09 ± 250.89 | -110.89 ± 210.48 | 0.952 | — |
| Lhip ext rate touchdown 1 toe-off 1 | Angular velocity | ANOVA | 0.12 | 0.885 | 0.006 | -93.67 ± 297.08 | -121.73 ± 276.46 | -70.50 ± 221.81 | 0.971 | — |
| Rthigh rom touchdown 1 touchdown 3 | Inter-segment coordination | ANOVA | 3.67 | 0.035 | 0.158 | 93.78 ± 3.90 | 91.03 ± 6.11 | 88.47 ± 5.40 | 0.339 | F/S* |
| Swing thigh rom | Inter-segment coordination | ANOVA | 3.60 | 0.037 | 0.156 | 95.14 ± 4.55 | 93.05 ± 6.90 | 89.29 ± 5.80 | 0.339 | F/S* |
| Swing thigh plus trunk min ft1 left | Inter-segment coordination | K-W | 5.03 | 0.081 | 0.078 | -25.21 ± 27.73 | -39.66 ± 23.63 | -4.53 ± 52.50 | 0.573 | — |
| Thigh plus trunk touchdown 2 left | Inter-segment coordination | K-W | 4.33 | 0.115 | 0.060 | -7.43 ± 12.00 | -15.28 ± 11.00 | 8.27 ± 44.18 | 0.704 | — |
| Lthigh rom touchdown 1 touchdown 3 | Inter-segment coordination | ANOVA | 2.20 | 0.124 | 0.102 | 97.68 ± 5.64 | 97.39 ± 5.07 | 93.75 ± 5.52 | 0.727 | — |
| Thigh plus trunk toe-off 1 left | Inter-segment coordination | K-W | 3.92 | 0.141 | 0.049 | -10.28 ± 38.78 | -27.78 ± 34.40 | 12.86 ± 60.56 | 0.728 | — |
| Thigh plus trunk toe-off 3 left | Inter-segment coordination | K-W | 3.60 | 0.165 | 0.041 | -9.00 ± 40.94 | -25.85 ± 35.14 | 13.31 ± 59.61 | 0.759 | — |
| Toeoff com dx toe-off 1 left | Inter-segment coordination | ANOVA | 1.86 | 0.170 | 0.087 | 0.20 ± 0.41 | -0.03 ± 0.38 | 0.23 ± 0.35 | 0.759 | — |
| Toeoff com dx toe-off 2 left | Inter-segment coordination | ANOVA | 1.71 | 0.195 | 0.081 | 0.12 ± 0.44 | 0.30 ± 0.34 | 0.02 ± 0.39 | 0.811 | — |
| Toeoff com dx toe-off 1 right | Inter-segment coordination | ANOVA | 1.69 | 0.199 | 0.080 | 0.11 ± 0.45 | 0.29 ± 0.36 | 0.01 ± 0.37 | 0.811 | — |
| Toeoff com dx toe-off 2 right | Inter-segment coordination | ANOVA | 1.43 | 0.252 | 0.068 | 0.20 ± 0.44 | -0.02 ± 0.37 | 0.21 ± 0.38 | 0.811 | — |
| Thigh plus trunk touchdown 3 right | Inter-segment coordination | K-W | 2.74 | 0.254 | 0.019 | 2.75 ± 29.70 | -8.38 ± 23.36 | -3.17 ± 22.02 | 0.811 | — |
| Thigh plus trunk toe-off 3 right | Inter-segment coordination | ANOVA | 1.36 | 0.267 | 0.065 | -2.14 ± 49.34 | 3.24 ± 47.83 | -23.61 ± 33.48 | 0.811 | — |
| Shank vs thigh toe-off 3 left | Inter-segment coordination | K-W | 2.55 | 0.279 | 0.014 | -126.08 ± 97.23 | -119.33 ± 78.42 | -75.62 ± 50.39 | 0.811 | — |
| Support foot vy touchdown 2 | Inter-segment coordination | K-W | 2.51 | 0.285 | 0.013 | 0.08 ± 1.68 | 0.36 ± 1.03 | 0.15 ± 0.81 | 0.811 | — |
| Lfoot vy touchdown 1 | Inter-segment coordination | ANOVA | 1.27 | 0.292 | 0.061 | -0.28 ± 1.61 | -1.00 ± 0.98 | -0.42 ± 1.13 | 0.811 | — |
| Lfoot vy touchdown 2 | Inter-segment coordination | K-W | 2.42 | 0.298 | 0.011 | -0.68 ± 1.50 | -0.11 ± 1.32 | -0.48 ± 0.95 | 0.811 | — |
| Support thigh rom | Inter-segment coordination | ANOVA | 1.23 | 0.303 | 0.059 | 96.32 ± 5.81 | 95.38 ± 5.85 | 92.94 ± 5.82 | 0.811 | — |
| Rfoot vy touchdown 1 | Inter-segment coordination | K-W | 2.32 | 0.313 | 0.008 | -0.84 ± 1.64 | -0.32 ± 1.20 | -0.72 ± 0.86 | 0.811 | — |
| Shank vs thigh toe-off 1 left | Inter-segment coordination | K-W | 2.25 | 0.324 | 0.006 | -110.42 ± 90.93 | -118.40 ± 76.94 | -75.31 ± 49.59 | 0.811 | — |
| Thigh plus trunk toe-off 1 right | Inter-segment coordination | ANOVA | 1.07 | 0.351 | 0.052 | -10.48 ± 47.16 | 2.99 ± 47.01 | -21.24 ± 32.31 | 0.811 | — |
| Thigh plus trunk toe-off 2 right | Inter-segment coordination | ANOVA | 1.05 | 0.358 | 0.051 | -3.86 ± 47.10 | -21.09 ± 36.87 | 0.81 ± 39.91 | 0.811 | — |
| Swing thigh minus shank min touchdown 1 toe-off 1 left | Inter-segment coordination | K-W | 1.91 | 0.386 | ≈0 | -127.68 ± 99.89 | -143.49 ± 81.60 | -91.79 ± 57.58 | 0.858 | — |
| Swing thigh plus trunk min ft2 right | Inter-segment coordination | ANOVA | 0.96 | 0.394 | 0.047 | -17.62 ± 42.33 | -32.89 ± 30.47 | -16.01 ± 31.71 | 0.861 | — |
| Shank vs thigh toe-off 2 left | Inter-segment coordination | ANOVA | 0.90 | 0.415 | 0.044 | -126.06 ± 73.62 | -104.81 ± 73.40 | -93.63 ± 41.36 | 0.874 | — |
| Thigh plus trunk touchdown 1 right | Inter-segment coordination | K-W | 1.75 | 0.416 | ≈0 | 1.27 ± 27.70 | -5.32 ± 22.72 | -0.87 ± 19.57 | 0.874 | — |
| Swing thigh minus shank min touchdown 3 toe-off 3 left | Inter-segment coordination | K-W | 1.71 | 0.425 | ≈0 | -145.53 ± 105.84 | -145.84 ± 83.04 | -95.42 ± 57.66 | 0.874 | — |
| Knee sep touchdown 2 | Inter-segment coordination | ANOVA | 0.81 | 0.453 | 0.040 | 0.03 ± 0.26 | -0.06 ± 0.19 | 0.03 ± 0.14 | 0.880 | — |
| Rfoot vy touchdown 2 | Inter-segment coordination | ANOVA | 0.78 | 0.464 | 0.039 | -0.30 ± 1.51 | -0.82 ± 1.00 | -0.31 ± 1.14 | 0.880 | — |
| Knee sep touchdown 1 | Inter-segment coordination | ANOVA | 0.76 | 0.477 | 0.037 | -0.05 ± 0.27 | 0.04 ± 0.17 | -0.04 ± 0.15 | 0.891 | — |
| Knee sep touchdown 3 | Inter-segment coordination | ANOVA | 0.68 | 0.513 | 0.034 | -0.02 ± 0.29 | 0.06 ± 0.18 | -0.03 ± 0.15 | 0.914 | — |
| Swing thigh minus shank min touchdown 2 toe-off 2 left | Inter-segment coordination | ANOVA | 0.58 | 0.564 | 0.029 | -144.04 ± 76.55 | -119.75 ± 76.52 | -120.59 ± 47.02 | 0.921 | — |
| Swing thigh minus shank min touchdown 1 toe-off 1 right | Inter-segment coordination | K-W | 1.01 | 0.603 | ≈0 | -129.48 ± 97.39 | -119.91 ± 100.42 | -144.89 ± 76.57 | 0.937 | — |
| Swing foot vy touchdown 2 | Inter-segment coordination | ANOVA | 0.51 | 0.604 | 0.026 | -1.06 ± 1.04 | -1.29 ± 0.70 | -0.94 ± 0.96 | 0.937 | — |
| Swing thigh plus trunk min ft1 right | Inter-segment coordination | K-W | 0.95 | 0.621 | ≈0 | -24.25 ± 38.82 | -14.29 ± 40.86 | -29.92 ± 25.79 | 0.937 | — |
| Shank vs thigh toe-off 1 right | Inter-segment coordination | K-W | 0.94 | 0.626 | ≈0 | -103.94 ± 91.58 | -101.21 ± 102.30 | -118.02 ± 68.12 | 0.937 | — |
| Swing thigh minus shank min touchdown 3 toe-off 3 right | Inter-segment coordination | ANOVA | 0.40 | 0.676 | 0.020 | -144.26 ± 105.69 | -121.79 ± 108.14 | -154.73 ± 77.97 | 0.937 | — |
| Thigh plus trunk toe-off 2 left | Inter-segment coordination | ANOVA | 0.36 | 0.703 | 0.018 | -14.93 ± 39.12 | -3.49 ± 33.78 | -11.02 ± 37.59 | 0.937 | — |
| Support com dx touchdown 3 left | Inter-segment coordination | ANOVA | 0.32 | 0.726 | 0.016 | 0.16 ± 0.38 | 0.09 ± 0.35 | 0.06 ± 0.32 | 0.937 | — |
| Support foot vy touchdown | Inter-segment coordination | ANOVA | 0.30 | 0.740 | 0.015 | -1.22 ± 1.09 | -1.41 ± 0.76 | -1.14 ± 0.85 | 0.945 | — |
| Swing thigh minus shank min touchdown 2 toe-off 2 right | Inter-segment coordination | K-W | 0.53 | 0.765 | ≈0 | -113.76 ± 115.09 | -137.78 ± 87.93 | -121.49 ± 71.30 | 0.946 | — |
| Shank vs thigh toe-off 2 right | Inter-segment coordination | K-W | 0.45 | 0.798 | ≈0 | -97.57 ± 107.22 | -116.51 ± 87.39 | -105.58 ± 67.09 | 0.946 | — |
| Thigh plus trunk touchdown 1 left | Inter-segment coordination | K-W | 0.45 | 0.800 | ≈0 | -11.30 ± 10.61 | -9.76 ± 9.51 | 2.05 ± 36.06 | 0.946 | — |
| Swing thigh plus trunk min ft2 left | Inter-segment coordination | ANOVA | 0.22 | 0.802 | 0.011 | -28.65 ± 29.14 | -23.95 ± 23.42 | -21.34 ± 35.56 | 0.946 | — |
| Shank vs thigh toe-off 3 right | Inter-segment coordination | ANOVA | 0.21 | 0.811 | 0.011 | -127.48 ± 106.33 | -106.87 ± 101.37 | -126.07 ± 67.00 | 0.946 | — |
| Support com dx touchdown 2 right | Inter-segment coordination | ANOVA | 0.18 | 0.835 | 0.009 | 0.15 ± 0.34 | 0.11 ± 0.36 | 0.07 ± 0.31 | 0.952 | — |
| Swing foot vy touchdown | Inter-segment coordination | K-W | 0.36 | 0.836 | ≈0 | 0.11 ± 1.82 | 0.08 ± 0.94 | -0.00 ± 0.80 | 0.952 | — |
| Thigh plus trunk touchdown 2 right | Inter-segment coordination | K-W | 0.31 | 0.856 | ≈0 | -1.32 ± 27.15 | 1.25 ± 30.64 | -6.40 ± 17.91 | 0.952 | — |
| Support com dx touchdown 3 right | Inter-segment coordination | K-W | 0.17 | 0.920 | ≈0 | 0.08 ± 0.42 | 0.06 ± 0.35 | 0.10 ± 0.31 | 0.982 | — |
| Support com dx touchdown 1 left | Inter-segment coordination | ANOVA | 0.06 | 0.938 | 0.003 | 0.12 ± 0.35 | 0.10 ± 0.33 | 0.08 ± 0.30 | 0.982 | — |
| Thigh plus trunk touchdown 3 left | Inter-segment coordination | K-W | 0.13 | 0.939 | ≈0 | -7.01 ± 12.15 | -8.55 ± 11.57 | 2.68 ± 36.06 | 0.982 | — |
| Support com dx touchdown 1 right | Inter-segment coordination | ANOVA | 0.05 | 0.949 | 0.003 | 0.09 ± 0.37 | 0.09 ± 0.34 | 0.12 ± 0.29 | 0.982 | — |
| Support com dx touchdown 2 left | Inter-segment coordination | ANOVA | 0.03 | 0.966 | 0.002 | 0.09 ± 0.36 | 0.08 ± 0.37 | 0.11 ± 0.31 | 0.982 | — |

*One-way ANOVA (η² reported) or Kruskal–Wallis test (K-W; ε² reported) between the three performance groups for each of 129 kinematic variables extracted via Sports2D/RTMPose pose estimation. FDR p-values were computed using the Benjamini–Hochberg procedure across the full set of 129 variables. Post-hoc pairwise comparisons (Tukey HSD or Dunn with Bonferroni): F/I = Faster vs Intermediate, F/S = Faster vs Slower, I/S = Intermediate vs Slower. * p < 0.05, ** p < 0.01, *** p < 0.001.*

**Supplementary Table S5. Breakdown of OpenSim-derived muscle–tendon kinematic parameters by muscle group and variable type.**

| **Category** | **Parameters tested** | **Raw p < 0.05** | **FDR-significant** |
| --- | --- | --- | --- |
| **By muscle group** |  |  |  |
| Hamstrings | 496 | 7 | 0 |
| Quadriceps | 496 | 9 | 0 |
| Gluteus maximus | 372 | 4 | 0 |
| Gluteus medius | 372 | 4 | 0 |
| Hip flexors | 248 | 3 | 0 |
| Triceps surae | 372 | 3 | 0 |
| Tibialis anterior | 124 | 1 | 0 |
| **Total** | **2480** | **31** | **0** |
| **By variable type** |  |  |  |
| Peak shortening velocity | 400 | 20 | 0 |
| Peak lengthening length/velocity | 800 | 5 | 0 |
| Muscle length ROM | 400 | 5 | 0 |
| Minimum length | 400 | 1 | 0 |
| Stance/swing MTU-length ratio | 120 | 0 | 0 |
| MTU-length index (left/right) | 240 | 0 | 0 |
| Phase-specific muscle length | 120 | 0 | 0 |

*Summary of between-group comparisons across 2,480 OpenSim-derived muscle–tendon kinematic parameters, organized by anatomical muscle group (top) and by parameter type (bottom). 'Raw p < 0.05' counts nominally significant results before multiple comparison correction (1.3% of all tests, below the 5% expected by chance). 'FDR-significant' shows the number of parameters surviving Benjamini–Hochberg correction at q < 0.05; zero parameters survived correction in any muscle group or variable type. All parameters are OpenSim-derived muscle–tendon unit lengths and contraction velocities sampled across the gait cycle and at foot-strike/toe-off events. No electromyography or static optimization was performed; variable-type labels denote MTU lengths, stance/swing MTU-length ratios, side-specific MTU-length indices, and contraction velocities only (length-based quantities, not neuromuscular estimates).*

**Supplementary Table S6. Sensitivity analysis of the mid-run muscle–tendon null: FDR significance and TOST equivalence by muscle group, with minimum detectable effect.**

| **Muscle group** | **Variables tested** | **Raw p < 0.05** | **FDR-significant** | **TOST-equivalent (n)** | **Equivalence established** |
| --- | --- | --- | --- | --- | --- |
| Hamstrings (BFlh, BFsh, SM, ST) | 496 | 7 | 0 | 0 | No |
| Quadriceps (RF, vasti) | 496 | 9 | 0 | 0 | No |
| Gluteals (max, med) | 744 | 8 | 0 | 0 | No |
| Hip flexors (psoas, iliacus) | 248 | 3 | 0 | 0 | No |
| Triceps surae (gastroc, soleus) | 372 | 3 | 0 | 0 | No |
| Other (e.g., tibialis anterior, adductors) | 124 | 1 | 0 | 0 | No |
| **ALL MTU (family total)** | **2480** | **31** | **0** | **0** | **No** |

*Mid-run (30–50 m) muscle–tendon (MTU) family. Significance from one-way ANOVA across the three performance groups; FDR computed across all 2,480 MTU variables (Benjamini–Hochberg). Equivalence assessed by two one-sided tests (TOST) comparing the faster (G1) and slower (G3) groups, with an a priori smaller-than-medium bound (Cohen's d = ±0.505, equivalent to η² < 0.06); a variable is declared equivalent at TOST p < 0.05. Minimum detectable effect (3-group ANOVA, n = 43, 80% power): Nominal α=0.05: Cohen f = 0.492 (η² = 0.195); Bonferroni α=0.05/2480 (MTU family): Cohen f = 0.948 (η² = 0.473); Bonferroni α=0.05/129 (kinematic family): Cohen f = 0.792 (η² = 0.385). No MTU variable in any muscle group reached FDR significance or established equivalence; the muscle–tendon null is therefore an underpowered, sensitivity-limited result rather than evidence of conservation/invariance.*
